# Enhancing patient care with AI agents: integrating advanced chatbot technologies for improved healthcare delivery

**DOI:** 10.1186/s12911-026-03694-9

**Published:** 2026-07-09

**Authors:** Yasmine Abu Adla, Ahmad El Hajj

**Affiliations:** American University of Beirut (AUB), Beirut, Lebanon

**Keywords:** AI-driven medical chatbot, AI agents, Knowledge graphs, RAG, GPT-4o, Healthcare technology, Healthcare automation

## Abstract

The integration of artificial intelligence (AI) in healthcare presents significant opportunities to enhance patient care and streamline medical workflows. However, challenges related to accuracy, reliability, and accessibility continue to limit the widespread adoption of AI-driven medical assistants. To the best of our knowledge, this is the first study to integrate multiple advanced AI techniques into a unified system designed to provide real-time, context-aware medical insights while ensuring accuracy, engagement, and interpretability. This study aims to develop an AI-powered medical agent capable of assisting both patients and healthcare professionals by generating informed medical responses, automating healthcare-related tasks, and improving patient interaction through interactive and reliable assistance. The methods employed in this research include Retrieval-Augmented Generation (RAG) for contextualized medical responses, the Wikipedia API for real-time knowledge retrieval, and knowledge graphs for mapping symptoms to potential diseases. Additionally, a symptom checker tool enables preliminary diagnosis and personalized health recommendations using a prompt-based system. The system was evaluated through automated performance assessments and expert reviews. The results demonstrate that the knowledge graph tool achieved 95% accuracy in medical query responses, showcasing its reliability in symptom-disease mapping. Additionally, sentiment analysis of patient interactions reached 97% accuracy, reinforcing the system’s ability to understand and respond empathetically. The Wikipedia-based retrieval system maintained an average response time of 10 seconds, ensuring real-time applicability. Furthermore, 100 medical experts rated the AI agent’s responses 4.6 for comprehensiveness, 4.5 for engagement, and 4.5 for empathy and tone on a 5-point scale. These findings suggest that integrating advanced AI techniques enhances the accuracy, responsiveness, and contextual relevance of AI-driven medical agents. This scalable and reliable system presents a viable solution to reduce healthcare workload, enhance patient engagement, and democratize access to trusted medical information, reinforcing its potential as a transformative tool in modern healthcare.

## Introduction

The rapid advancement of artificial intelligence has brought transformative changes across various industries, with healthcare being one of the most promising beneficiaries. AI technologies have the potential to revolutionize patient care by providing innovative solutions that can alleviate the challenges faced by healthcare systems, such as workforce shortages, rising costs, and the need for continuous patient engagement Topol [1]. In this context, AI-driven chatbots, or AI agents, are emerging as vital tools capable of supporting both patients and healthcare professionals by automating routine tasks, enhancing communication, and facilitating access to medical information Joshi and Sharma [2].

Furthermore, recent studies have demonstrated the effectiveness of explainable AI in healthcare applications, enabling more interpretable decision-making processes for critical tasks such as lung cancer detection, breast cancer classification, and cardiac disease prediction [3, 4, 5]. These advancements in AI-driven diagnostics and predictive modeling highlight the growing role of AI in enhancing medical accuracy, improving patient outcomes, and supporting clinical decision-making Rasool et al. [6], Wani et al. [5].

AI chatbots have been deployed in healthcare for a range of purposes, including mental health support Miner et al. [7], Molli [8], Casu et al. [9], symptom checking, and appointment scheduling Cao and Wang [10]. These applications demonstrate the potential of AI-driven tools to empower patients and streamline healthcare services. Moreover, studies have shown that AI chatbots can significantly reduce the burden on healthcare providers, allowing them to focus on more critical tasks that require human expertise Bates and Sheikh [11], Molli [8], Casu et al. [9].

Natural language processing (NLP) is a fundamental component of AI chatbots, enabling them to understand and respond to patient queries in a conversational manner. Recent advancements in NLP, such as Retrieval-Augmented Generation, have further improved the ability of AI agents to retrieve relevant information and provide accurate, context-sensitive responses Lewis et al. [12]. RAG integrates the strengths of information retrieval and natural language generation, making it a powerful tool for medical chatbots that require real-time access to vast amounts of health-related information.

In addition to RAG, knowledge graphs are being used to enhance the functionality of AI agents by mapping patient symptoms to potential diseases Hogan et al. [13]. Knowledge graphs provide a structured representation of medical knowledge, enabling AI agents to infer relationships between symptoms, conditions, and treatments. This capability is particularly useful for developing sophisticated symptom checkers, which can offer users potential diagnoses and health advice based on their symptoms Zhou et al. [14].

Despite the advancements in AI-driven healthcare solutions, traditional healthcare chatbots face several critical limitations that hinder their effectiveness in real-world medical applications Fadhil and Schiavo [15], Xue et al. [16]. One major limitation is the lack of context awareness. Current systems often struggle with contextual reasoning and dynamic information retrieval, leading to inaccurate and generic responses. Most conventional healthcare chatbots operate using rule-based or retrieval-based models, which lack the ability to understand user intent beyond pre-defined scenarios. This rigidity prevents them from dynamically adjusting to complex medical cases that require deeper contextual reasoning.

Another significant shortcoming is their limited medical information synthesis. Regular chatbots struggle to integrate and synthesize diverse medical knowledge from multiple sources, such as research articles, patient histories, and real-time clinical updates. As a result, they fail to provide comprehensive, evidence-based recommendations, leading to oversimplified or inaccurate responses Laymouna et al. [17].

Additionally, many chatbots rely on static and predefined knowledge bases. These fixed medical databases do not update dynamically with new treatments, evolving disease patterns, or emerging research. This restricts their ability to support real-time, data-driven decision-making for healthcare professionals and patients Fadhil and Schiavo [15].

Limited personalization is another drawback of traditional healthcare chatbots. They often deliver generic responses that do not take into account individual patient history, symptoms, or medical background. This lack of personalization reduces their ability to offer tailored guidance and impacts user trust in AI-assisted healthcare Seitz [18].

Furthermore, conventional chatbots suffer from a lack of interpretability and transparency. They typically do not explain how they arrive at their conclusions, making their responses opaque to both medical professionals and patients. This black-box nature raises concerns regarding trust, accuracy, and clinical reliability, preventing widespread adoption in critical healthcare settings Kakolu and Faheem [19].

To address these limitations, we propose an AI-driven medical agent designed to enhance patient care and support healthcare professionals by integrating advanced AI techniques. This study specifically tackles the challenge of delivering accurate, context-aware healthcare advice by incorporating knowledge graphs and RAG to improve decision-making and real-time information retrieval. By leveraging these technologies, our system delivers dynamic, evidence-based insights, ensuring that medical responses remain contextually relevant and aligned with the latest advancements in healthcare.

A key component of our approach is RAG, which enables real-time information retrieval from updated medical sources, ensuring that responses are both accurate and evidence-based. Additionally, the integration of knowledge graphs enhances structured, context-aware medical reasoning, allowing for more precise disease-symptom mapping and personalized recommendations tailored to individual patient needs.

Our AI agent also incorporates a symptom checker tool, which utilizes a prompt-based AI model to dynamically evaluate patient inputs. By considering real-time contextual information, this tool enhances the accuracy of preliminary diagnoses and health advice, offering users a more responsive and intelligent healthcare assistant.

By integrating these state-of-the-art techniques, our AI agent surpasses traditional chatbots by dynamically adapting to real-time medical advancements, providing context-aware insights, and ensuring transparency in AI-driven decision-making. This advancement bridges the gap between automation and trustworthy AI-assisted healthcare, offering a scalable, adaptive, and reliable solution for modern medical applications.

To ensure robustness, reliability, and contextual relevance, our AI agent undergoes comprehensive evaluation, incorporating both automated performance assessments and expert human reviews. Through these rigorous validation methods, the system is designed to deliver accurate, engaging, and medically sound responses. Ultimately, this study demonstrates the potential of AI-driven medical agents to enhance patient care, streamline medical consultations, foster patient engagement, and contribute to better health outcomes while reducing the workload on healthcare professionals.

### Contributions

This paper presents a novel AI medical agent that integrates knowledge graphs for contextual understanding and RAG for real-time data retrieval. The key contributions of this research are as follows:

- **Development of an AI-Powered Medical Assistant:** This research introduces an AI-driven medical chatbot capable of providing real-time, personalized, and contextually relevant healthcare advice, surpassing traditional medical chatbots in diagnostic accuracy and user engagement.
- **Integration of Knowledge Graphs and RAG for Enhanced Query Handling:** The chatbot employs a knowledge graph to map relationships between diseases, symptoms, and treatments, enabling comprehensive and accurate medical responses. The incorporation of RAG allows for real-time retrieval of updated medical knowledge, ensuring accurate and relevant healthcare information.
- **Implementation of a Symptom Checker for Preliminary Diagnosis:** The system includes an advanced prompt-based AI model that enables preliminary diagnosis and health recommendations, enhancing the chatbot’s ability to guide users in medical decision-making.
- **High Accuracy in Medical Query Responses:** The knowledge graph-powered chatbot demonstrated a 95% accuracy rate in handling healthcare-related queries, improving reliability and precision in AI-assisted diagnostics.
- **Sentiment Analysis for User Engagement:** Patient interaction analysis achieved a 97% accuracy rate in sentiment detection, ensuring empathetic and contextually appropriate responses to improve user experience and trust.
- **Real-Time Medical Knowledge Retrieval:** The Wikipedia-based retrieval system maintained an average response time of 10 seconds, demonstrating its capability for real-time applicability in medical consultations.
- **Personalized Healthcare Recommendations:** The chatbot utilizes user-specific historical data and preferences to generate tailored healthcare advice, improving patient satisfaction and engagement.
- **Advanced Healthcare Facility Recommendation System:** A recommendation system leveraging RAG and patient reviews guides users in selecting optimal healthcare facilities based on quality, proximity, and patient-reported experiences.
- **Comprehensive System Architecture for AI-Driven Healthcare:** The proposed system integrates Wikipedia API, knowledge graphs, symptom checkers, and intelligent query processing, providing a scalable and adaptable architecture for AI medical assistants.
- **Error Handling for Enhanced Reliability:** A fallback mechanism ensures responses are always provided, even when user queries are incomplete or ambiguous, enhancing system robustness in real-world applications.

These contributions demonstrate the potential of our AI-driven medical chatbot to transform patient care by providing accurate, contextually relevant information, fostering patient engagement, and supporting healthcare professionals in decision-making. Our comprehensive integration of state-of-the-art AI technologies offers a valuable tool for improving the quality of healthcare delivery and democratizing access to medical information.

The remainder of this paper is structured as follows: Sect. 2 presents a review of related work, highlighting recent advancements in the use of AI chatbots in healthcare, including applications in mental health support, symptom checking, and appointment scheduling. Section 3 details the methodology, including the design and integration of advanced AI components such as the Wikipedia API, RAG, and knowledge graphs. Section 4 focuses on enhancing GPT for healthcare interactions by outlining the fine-tuning process and evaluating the model’s performance. Section 5 describes how the AI medical agent is empowered through various tools, including a symptom checker and real-time information retrieval mechanisms. Section 6 introduces the comprehensive system architecture, explaining how different components interact to provide accurate and context-aware responses. Section 7 presents the results and discussion, evaluating the system’s effectiveness and detailing insights from various test scenarios. Finally, Sect. 8 provides the conclusion and discusses potential future work.

## Related work

### Medical chatbots and AI integration

The rapid advancement of AI technologies has revolutionized various sectors, including healthcare. Among these innovations, medical chatbots have emerged as transformative tools with the potential to significantly enhance patient care and support medical professionals. Medical chatbots leverage AI to simulate human-like interactions, offering patients immediate assistance and providing healthcare professionals with support in routine tasks. These AI-driven agents hold promise for reducing healthcare costs, improving patient engagement, and delivering consistent medical information to patients.

Medical chatbots integrate a range of advanced AI components, including knowledge graphs, RAG, logical inference, and fine-tuned conversational models. Knowledge graphs are particularly powerful for structuring and interrelating medical information, enabling chatbots to understand complex relationships between diseases, symptoms, treatments, and medications. By utilizing established medical databases, such as Unified Medical Language System (UMLS) Clinfowiki [20] and Systematized Nomenclature of Medicine Clinical Terms (SNOMED CT) National Library of Medicine [21], knowledge graphs create a structured framework that significantly enhances a chatbot’s diagnostic accuracy and reliability. This structured approach has been shown to be highly beneficial, particularly in handling intricate medical scenarios where accurate and contextually relevant information is crucial for effective healthcare outcomes.

### Large language models for healthcare

Recent studies have explored a variety of LLM applications in healthcare, such as medical question-answering, clinical decision support, and patient interaction. For example, the MedPaLM2 model has been found to outperform previous versions in medical question answering, achieving significantly higher accuracy in practical applications Karabacak and Margetis [22], Wu et al. [23]. ChatGPT and related LLMs have also been utilized for document generation in electronic medical records and have been proposed for use in clinical decision-making contexts Chen et al. [24], Haemmerli et al. [25]. Zhou et al. highlighted the development and use of an LLM specifically aimed at providing clinical guidance in diagnostic settings Zhou et al. [26]. The recent ClinicBench framework further evaluated the clinical capabilities of LLMs, emphasizing their role in improving clinical problem-solving tasks, including treatment recommendations and patient education Li et al. [27].

Additionally, several recent reviews have highlighted the critical importance of addressing biases and hallucinations in LLMs, which are challenges that need to be resolved for more trustworthy healthcare applications. For example, a comprehensive review by Zhao et al. provides insights into debiasing techniques and solutions for hallucinations in LLM-generated content Zhao et al. [28]. This helps to ensure that healthcare systems employing LLMs maintain accuracy and fairness.

Recent studies have also highlighted the growing use of AI in pharmaceutical research and diagnostics. AI has become instrumental in accelerating drug discovery and improving disease diagnosis accuracy using various AI techniques such as machine learning and deep learning Ma et al. [29], Owasis and Nithya [30]. In addition, systematic literature reviews have provided extensive insights into the use of AI for disease diagnostics and impact assessments in healthcare, highlighting the potential and challenges involved in leveraging AI technologies for clinical decision support Hassanzadeh and Atyabi [31], Owasis and Nithya [30].

Another key technology used in medical chatbots is RAG, which combines the strengths of information retrieval and text generation. In healthcare contexts, RAG models allow chatbots to provide responses that are not only coherent but also grounded in reliable medical knowledge. By retrieving relevant documents from large corpora and generating responses based on this retrieved content, RAG-enhanced chatbots can deliver more accurate and informative responses compared to traditional generative models. This ensures that the information provided to users is of high quality, evidence-based, and reflective of the latest medical knowledge Perception and PRIME [32], Wang et al. [33].

Logical inference also plays a critical role in advancing the capabilities of medical chatbots. By integrating inference mechanisms, chatbots can reason about medical information and draw conclusions based on the available evidence, thereby handling more complex diagnostic queries and providing personalized treatment advice. This enhances the chatbot’s functionality in offering comprehensive clinical decision support Longo et al. [34], Schulte [35]. Additionally, the use of fine-tuned conversational models, trained on extensive medical datasets, has allowed for the development of more sophisticated agents capable of nuanced, context-aware interactions. These models enhance the chatbot’s ability to understand specific medical inquiries and respond in a way that fosters meaningful patient engagement, a key factor in improving healthcare experiences.

Several studies have also discussed the role of conversational AI models like MOSS and ChatGPT in transforming healthcare interactions. These models can understand and generate responses to diverse healthcare queries, effectively supporting both patients and healthcare professionals. The MOSS open conversational LLM, for example, is designed to facilitate multi-turn conversations and has shown promise in healthcare contexts Zeng et al. [36]. Similarly, a comprehensive study on ChatGPT discusses its role in enhancing user engagement and providing a reliable conversational partner in healthcare Cheyer et al. [37].

Robots and autonomous agents are increasingly playing critical roles in healthcare, from surgical assistance to rehabilitation and autonomous diagnostics. Surgical robots, such as the da Vinci Surgical System, are widely used to perform minimally invasive surgeries, while rehabilitation robots like Lokomat and HAL are used to assist in patient rehabilitation Troiano and Nolan [38]. Autonomous agents have also been leveraged in multi-agent systems to coordinate healthcare activities, optimize resource allocation, and improve overall care efficiency Kerkmann and Rothe [39].

### Challenges in AI-driven healthcare chatbots

Despite these advancements, several challenges remain that must be addressed to fully harness the potential of medical chatbots. Ensuring the accuracy and reliability of chatbot-generated medical advice is paramount. This necessitates rigorous validation, ongoing updates to the underlying medical knowledge, and adherence to strict ethical guidelines. Data privacy, ethical considerations, and regulatory compliance also present significant barriers to the widespread adoption of these technologies in healthcare settings Meyer et al. [40], Milano et al. [41], Raza et al. [42], Zhou et al. [43, 44], Zeng et al. [45]. Moreover, the personalization of medical advice remains limited, with many existing chatbots providing general information without considering individual user preferences or specific circumstances. Personalized advice is particularly important in cases where patient history and context are critical for delivering reliable recommendations. Addressing these limitations requires the integration of more sophisticated algorithms and adaptive learning techniques, allowing chatbots to tailor responses more closely to individual patient needs Chow et al. [46], Sarraju et al. [44].

As summarized in Table 1, existing studies largely concentrate on preliminary diagnostic support and enhancing patient engagement through basic interaction. Current systems are primarily based on simple information retrieval techniques, which significantly limit their decision-making capabilities. These systems lack the ability to synthesize information from multiple sources to derive actionable insights, primarily due to the absence of integrated inference mechanisms and advanced knowledge frameworks.

**Table 1:** Comparison of existing and proposed medical chatbot studies

Aspect	Existing Studies	Proposed Study	Addressed Gap
Functionality Scope	Focus on preliminary diagnoses and basic patient engagement, providing generic information with limited data integration and computational capabilities Safi et al. [47], Chen et al. [24], Haemmerli et al. [25], Karabacak and Margetis [22], Wu et al. [23].	Expands functionality by integrating multiple data sources, including real-time data, and utilizing advanced inference mechanisms to generate personalized and actionable healthcare insights and recommendations Kerkmann and Rothe [39], Owasis and Nithya [30], Li et al. [27].	Overcomes the limitation of basic engagement by incorporating decision-making capabilities, leveraging diverse data sources, and providing holistic insights for better healthcare navigation and treatment options.
Technology Integration	Utilize isolated components, such as knowledge graphs, RAG, and fine-tuned models, but lack comprehensive integration, limiting their ability to generate complex, context-aware responses. They struggle to incorporate external data effectively Li et al. [48], Ma et al. [29], Zhao et al. [28].	Integrates advanced technologies, such as knowledge graphs, RAG, logical inference, and fine-tuned conversational models, into a unified framework. This allows to infer complex relationships and dynamically update its responses based on new information Kerkmann and Rothe [39], Perception and PRIME (2024), Wang et al. [33].	Addresses the lack of integration by creating a unified, context-aware AI system capable of advanced reasoning and complex decision-making processes for improved responses.
Personalization	Rule-based and constrained by limited training data. Responses are static, using predefined decision trees that fail to adapt to the variability of individual patient needs and preferences, resulting in generic and less impactful interactions Chow et al. [46], Sarraju et al. [44].	Incorporates adaptive learning techniques, enabling personalization based on user-specific data, such as preferences, historical interactions, and real-time feedback. This ensures more relevant and effective healthcare interactions Hassanzadeh and Atyabi [31], Milano et al. [41], Raza et al. [42].	Overcomes static personalization by using adaptive models that continuously learn from interactions, leading to improved responsiveness and tailored care for patients.
Data Sources	Rely on structured, static datasets, which limits adaptability and prevents the use of patient-generated data or feedback loops. Information provided may be outdated or irrelevant to the user’s specific context Ma et al. [29], Newbury et al. [49].	Leverages a broader range of data sources, including user-generated content, reviews, and real-time patient feedback, which allows for more dynamic, context-aware responses based on the latest information Abd-Alrazaq et al. [50], Macdonald et al. [51].	Enhances adaptability and relevance by integrating both structured and unstructured data, including real-time inputs, thereby improving the quality and accuracy of advice provided.
Clinical Decision Support	Simple information retrieval with basic guidance, lacking logical inference capabilities. This limits their ability to provide nuanced clinical recommendations tailored to specific patient scenarios Cheyer et al. [37], Hassanzadeh and Atyabi [31], Wang et al. [33].	Incorporates logical inference to analyze patient data, infer medical conditions, and recommend personalized treatment plans. It integrates clinical guidelines and patient data for comprehensive decision support in complex scenarios Longo et al. [34], Owasis and Nithya [30], Schulte [35].	Provides enhanced decision support by using logical inference and advanced reasoning, enabling the system to offer sophisticated, personalized recommendations based on a complete patient profile.
User Interaction	Basic conversational interactions with limited memory of past interactions and no capability for multi-turn dialogue, resulting in disjointed and repetitive conversations that negatively impact patient engagement Safi et al. [47], Troiano and Nolan [38].	Enhanced conversational models that retain and utilize the context of previous interactions, ensuring coherent, relevant, and context-aware responses Cheyer et al. [37], Zeng et al. [36].	Addresses context retention limitations by employing advanced memory mechanisms, providing a seamless user experience, and fostering continuous and coherent interactions that maintain engagement and trust.

A critical limitation of current AI-driven medical chatbots is their inability to process and correlate multimodal medical data, such as electronic health records (EHRs), real-time wearable sensor data, lab results, and historical patient information. Most existing systems rely on pre-defined rule-based approaches or static databases, failing to dynamically adapt recommendations based on a holistic view of the patient’s condition. This results in generic, one-size-fits-all responses that do not capture individualized health contexts or evolving medical conditions over time.

Furthermore, the lack of a robust knowledge graph means that relationships between different medical entities—such as symptoms, diseases, treatments, and patient-specific attributes—are not effectively captured. Traditional knowledge graphs used in healthcare chatbots are often manually curated, making them prone to outdated information and missing critical relationships. These limitations restrict chatbots from performing context-aware reasoning, an essential capability for complex decision-making in clinical applications.

Additionally, existing models fail to incorporate causal inference and reasoning-based decision support, which are crucial for predicting disease progression, identifying potential complications, and tailoring personalized treatment recommendations. Without these capabilities, chatbots cannot weigh multiple patient-specific factors to recommend contextually relevant, evidence-based interventions. The absence of these advanced inference mechanisms significantly reduces the chatbot’s reliability in real-world medical decision-making scenarios.

Another major gap is the inability of current chatbots to integrate real-time, continuously evolving medical knowledge. Given the rapid advancements in medicine, treatment protocols and best practices frequently change. However, most AI-driven medical chatbots rely on pre-trained models or static knowledge bases, limiting their ability to dynamically update responses based on the latest clinical guidelines, ongoing medical trials, and emerging research findings. This constraint poses a significant challenge in critical care applications, where up-to-date medical knowledge can be a determining factor in patient outcomes.

Moreover, existing healthcare chatbots often lack adaptive learning mechanisms, meaning they do not improve over time based on user interactions. Unlike human clinicians who refine their expertise through experience, chatbots fail to learn from past cases, patient feedback, or real-world medical trends, leading to repetitive errors and suboptimal recommendations. Incorporating continuous learning and reinforcement learning techniques could enhance chatbot reliability and enable personalized, evolving healthcare interactions.

Addressing these limitations requires a paradigm shift in chatbot architecture, moving beyond simple retrieval-based models to integrated AI frameworks that combine real-time data processing, knowledge graphs, causal reasoning, and adaptive learning techniques. Future advancements should focus on multi-agent AI systems, where specialized AI components collaboratively analyze patient data, retrieve relevant knowledge, and generate informed medical recommendations. Without these improvements, AI-driven healthcare chatbots will remain limited in scope, accuracy, and practical usability in clinical environments.

Therefore, the proposed study aims to significantly extend the capabilities of medical chatbots by integrating advanced decision-making features and delivering actionable healthcare advice. By incorporating cutting-edge technologies such as knowledge graphs, RAG, logical inference, and fine-tuned conversational models, this approach is designed to offer a more holistic and sophisticated range of diagnostic and advisory services.

The use of knowledge graphs is central to enhancing the chatbot’s reasoning capabilities. Knowledge graphs provide a structured representation of medical information, capturing complex interrelationships between diseases, treatments, symptoms, and medications. However, a significant limitation of knowledge graphs is their inherent incompleteness and biases, affecting their utility in critical applications like healthcare Zeng et al. [52]. By using established ontologies, such as the UMLS or SNOMED CT, the chatbot can create a rich semantic context that significantly enhances its diagnostic accuracy. The combination of knowledge graphs with RAG enables the chatbot to leverage both structured knowledge and unstructured information from large medical corpora, providing responses that are both evidence-based and context-specific. However, challenges like redundant content retrieval and issues with semantic integrity in fine-grained retrieval units are notable drawbacks of using RAG in complex systems Gao and Smith [53]. Additionally, constructing reliable healthcare knowledge graphs requires effective entity extraction and relation linking, ensuring semantic accuracy across diverse medical domains Abu-Salih et al. [54].

Additionally, to the best of our knowledge, this is the first time a mix of AI agents has been integrated in such a manner to address different specialized aspects of healthcare comprehensively. The proposed system integrates a mixture of AI agents, each specialized in a different aspect of healthcare. For example, one agent may focus on analyzing patient-specific medical history, while another handles real-time retrieval of medical literature, and yet another performs logical inference based on clinical guidelines. This multi-agent approach enables parallel processing and specialization, thereby increasing the efficiency and reliability of the chatbot’s responses. Each agent contributes its specialized knowledge, which is then combined to generate a holistic and comprehensive recommendation for the user. This framework is instrumental in breaking down complex medical problems into smaller, more manageable components, each handled by an expert agent, and then aggregating these insights for a well-rounded decision.

Dynamic querying plays a crucial role in ensuring that the chatbot’s responses are current and accurate. Unlike static systems that rely on pre-existing datasets that may become outdated, dynamic querying allows the proposed system to access real-time information. This means the chatbot can retrieve the latest medical guidelines, newly published research, and even patient-generated data, such as wearable device metrics, ensuring that the advice provided is always up-to-date and relevant. For instance, the chatbot can perform dynamic queries against clinical trial databases to determine the most recent treatment protocols for a specific condition. By dynamically querying both structured and unstructured data sources, the chatbot maintains a high level of adaptability and accuracy.

Moreover, by leveraging a knowledge graph combined with logical inference, the proposed system is capable of advanced contextual reasoning. For instance, when presented with a patient’s symptoms and medical history, the chatbot can navigate the graph to identify possible comorbidities and recommend a customized treatment plan. This capability significantly outperforms traditional retrieval-based systems that often fail to consider the broader medical context, such as overlapping symptoms and interactions between concurrent treatments.

The integration of these advanced tools—knowledge graphs, RAG, logical inference, and a mixture of specialized AI agents—provides a comprehensive and dynamic decision-support environment. The proposed system is not only capable of responding to user queries but can also proactively guide patients through decision pathways, recommend next steps, and ensure follow-up actions are personalized and evidence-based. These enhancements have the potential to greatly improve both the quality of care and the level of patient engagement, ultimately leading to more accurate diagnoses and better health outcomes.

### Comparative analysis of existing medical chatbots

The landscape of AI-driven healthcare chatbots has seen significant advancements, with various systems offering specialized functionalities. However, these solutions often exhibit limitations in their technology, data integration, and overall aims. As shown in Table 2, we compare the key differences in technology, data integration, and aims between our proposed solution and other AI-driven healthcare chatbots.

**Table 2:** Comparison of AI-Driven healthcare chatbots

Chatbot	Data Source	Technology Used	Functionality	Aim	Data Integration	Technical Details
MedPaLM (Singhal et al. [55])	Large corpus of medical literature and clinical data.	LLMs developed by Google and DeepMind.	Provides medical information and answers health-related questions.	Offer accurate medical responses and assist in patient education.	Integrates with medical databases and knowledge bases for up-to-date information.	Advanced NLP techniques to understand and generate human-like text.
Ada Health (Jungmann et al. [56])	Curated medical knowledge base developed over 10 years.	Probabilistic reasoning and AI algorithms.	Assesses user symptoms and provides possible diagnoses.	Help users understand their symptoms and suggest potential conditions.	Integrates with electronic health records and health systems for personalized assessments.	Combines probabilistic reasoning with a medical knowledge base.
Abridge (Konam and Rao [57])	Patient-clinician conversations.	AI to automate clinical documentation.	Transcribes and summarizes medical conversations for clinicians.	Reduce administrative burdens on healthcare providers.	Integrates with healthcare systems to streamline documentation processes.	Focuses on speech recognition and NLP to accurately capture medical dialogues.
Babylon Health (Schulz et al. [58])	Curated medical knowledge, patient history, and self-reported data.	AI algorithms, symptom checker, virtual consultations.	Provides medical consultations based on personal data and medical history.	Offer quick medical advice and virtual consultations.	Integrates with personal medical data, EHRs, and medical knowledge.	AI to interpret symptoms, offer diagnoses, and assist in virtual consultations.
OneRemission (OneRemission [59])	Cancer patient data, health reports, medical history.	AI, sentiment analysis, and health data interpretation.	Provides information, support, and emotional engagement to cancer patients.	Provide personalized support and guidance to cancer patients.	Integrates with patient data and medical reports for personalized insights.	Uses sentiment analysis to enhance emotional support and decision-making.
Our Chatbot	Wikipedia API, Knowledge Graphs, RAG.	RAG, Knowledge Graphs, Symptom Checker tool.	Assists patients and healthcare professionals by providing medical responses and improving engagement.	Reduce healthcare workload, enhance patient engagement, and democratize access to reliable medical information.	Integrates with real-time knowledge retrieval systems, Knowledge Graphs, and Symptom Checkers.	Advanced NLP techniques, RAG, to ensure accurate, context-aware responses.

By examining these key factors, it becomes evident how our approach addresses gaps in the current technologies and offers a more robust, dynamic, and context-aware solution. The current state-of-the-art AI-driven healthcare chatbots like MedPaLM and Ada Heath primarily rely on LLMs to generate medical responses Jungmann et al. [56], Singhal et al. [55]. MedPaLM, developed by Google and DeepMind, leverages LLMs trained on vast medical literature. While it offers detailed medical information, its dependence on pre-trained databases limits its capacity to provide real-time responses or adapt to new medical knowledge. Similarly,Ada Health uses a bounded LLM focused on accuracy through peer-reviewed medical journals, but it lacks the ability to interact dynamically with user-specific medical contexts or perform real-time knowledge retrieval.

In contrast, our chatbot incorporates RAG in combination with knowledge graphs to generate context-aware medical responses. This integration of real-time knowledge retrieval using the Wikipedia API ensures that the system is constantly updated with the latest information, allowing it to respond accurately to user queries. Furthermore, unlike other chatbots that rely heavily on probabilistic reasoning, our solution uses knowledge graphs to map symptoms to potential diseases with higher precision, facilitating more personalized and accurate assessments.

Most existing chatbots, such as Ada Health and Babylon Health, leverage curated medical knowledge bases or user interactions to assess symptoms and provide diagnoses. Ada Health, for instance, uses a curated knowledge base developed over a decade, and Babylon Health combines conversational AI with medical guidelines for virtual consultations and symptom checking. However, these systems often rely on static databases or narrow functionality, such as symptom-based assessments or administrative tasks, limiting their ability to adapt to new conditions or provide holistic healthcare support.

Our chatbot, on the other hand, integrates a variety of data sources, including real-time knowledge retrieval systems (e.g., Wikipedia API), knowledge graphs, and a symptom checker tool that assists in both diagnosis and personalized health recommendations. The combination of these data sources enables our chatbot to not only respond to medical queries but also to provide preliminary diagnoses based on user-reported symptoms, offering a more comprehensive and dynamic healthcare experience.

The aims of existing healthcare chatbots vary, with some focusing on reducing administrative workload (e.g., Abridge automates clinical documentation) and others on enhancing patient engagement or providing symptom assessments (e.g., Ada Health and Babylon Health). While these systems address specific needs, they are limited in scope and functionality. Abridge, for example, focuses on automating documentation and does not provide real-time diagnostic support or interactive engagement with users.

In contrast, our chatbot aims to reduce healthcare workload, enhance patient engagement, and democratize access to reliable medical information. By leveraging advanced NLP techniques and combining them with real-time data sources, context-aware symptom analysis, and interactive health recommendations, our chatbot offers a more sophisticated and scalable solution for modern healthcare needs. Additionally, the integration of symptom checkers and knowledge graphs enables our chatbot to deliver high accuracy in disease mapping, a critical advantage over systems relying solely on probabilistic reasoning or static knowledge bases.

## Methodology

The methodology employed in this study follows a structured workflow designed to ensure a seamless integration of various AI components, from data preprocessing to the final implementation of the user interface. The comprehensive workflow begins with data collection and preprocessing, ensuring that datasets are cleaned and normalized to enhance the performance of the chatbot model. The subsequent stages involve the training and fine-tuning of the model, incorporating advanced AI techniques, such as RAG and knowledge graphs, to enrich the chatbot’s capabilities. Finally, the model is integrated into a user-friendly interface that allows end-users to interact with the chatbot effectively.

**Fig. 1:**
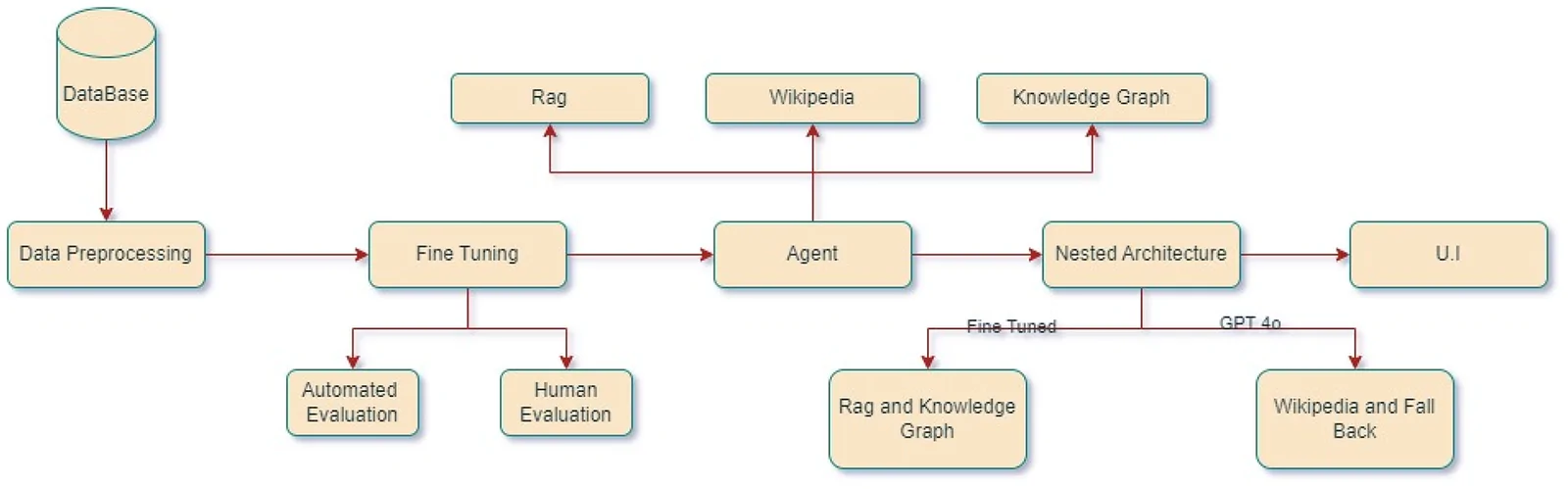
Detailed workflow of the project: From data preprocessing to user interface implementation

The flowchart in Figure 1 illustrates the key stages of this workflow, highlighting critical milestones and decision points, including data processing, model training, evaluation, and system deployment. Each phase was meticulously designed to improve the chatbot’s diagnostic and advisory capabilities, ultimately contributing to a better healthcare experience for users. The structured approach enabled us to manage the complexity of integrating multiple AI technologies while maintaining the robustness and reliability of the system.

### Dataset Description

To enhance the functionality and performance of our medical chatbot, several datasets were utilized, each serving a distinct purpose. These datasets enabled the development of a robust system capable of delivering informed, personalized, and contextually relevant medical advice to users. Table 3 provides a summary of the datasets, highlighting their characteristics and contributions to the study.

**Table 3:** Summary of datasets used in the study

Dataset	Description	Purpose
Hospital Reviews Dataset	Contains 1,005 records with attributes such as review ID, visit ID, review text, physician name, hospital name, and patient name. Focuses on evaluating patient experiences, including quality of care, facilities, and staff.	Utilized in the RAG model to provide contextually relevant responses based on real patient feedback.
MTS-Dialog Dataset	Composed of training (1,201 entries), validation (100 entries), and testing (200 entries) subsets. Each entry includes demographics (e.g., age, ethnicity) and dialogues representing doctor-patient interactions.	Fine-tuned the model to generate accurate, context-aware responses, facilitating meaningful medical interactions Abachaa [60].
Disease Symptoms and Patient Profile Dataset	Contains 349 cases linking diseases, symptoms (e.g., fever, cough, fatigue), and patient demographics, along with unique patient identifiers for individual tracking.	Used to construct a knowledge graph, facilitating predictive healthcare analytics and enhancing diagnostic capabilities Anonymous [61].

#### Ensuring data security and privacy compliance

The integration of sensitive health data within our AI system necessitates stringent security measures to ensure data protection and compliance with regulatory standards, such **HIPAA** Hash et al. [62]. To achieve this, we have implemented a multi-layered security approach that safeguards patient information from unauthorized access, data breaches, and privacy violations.

1. **Data Encryption:** All data is encrypted both at rest and in transit using industry-standard encryption protocols, ensuring that unauthorized entities cannot access or manipulate sensitive information.
2. **Strict Access Control:** We employ role-based access controls and multi-factor authentication to limit system access to only authorized personnel. This minimizes potential exposure to confidential patient data.
3. **Anonymization and De-Identification:** Whenever possible, personally identifiable information is removed or anonymized before being processed by the AI system. This approach reduces risks associated with data re-identification and ensures compliance with privacy laws.
4. **Secure Storage and Data Transfer:** The system is hosted on GDPR-compliant and HIPAA-certified cloud infrastructure, ensuring that data storage and transfers meet the highest security standards. Regular security audits and penetration testing are conducted to identify and mitigate vulnerabilities.
5. **Audit Logs and Real-Time Monitoring:** Continuous logging and monitoring mechanisms are in place to track system activity. These logs help detect unauthorized access attempts and potential security threats, enabling rapid response to mitigate risks.
6. **Compliance and Training:** Team members involved in handling sensitive data undergo regular training on best practices for data privacy, security protocols, and compliance requirements. This ensures that the system maintains high-security standards across all operational levels.

### Dataset pre-processing

Effective data preprocessing is critical to ensure the datasets are clean, consistent, and ready for model training, particularly in enhancing the Large Language Model (LLM) for medical dialogues. The following preprocessing steps were undertaken:

**Data Cleansing:** Initially, all unique characters in the datasets were identified to detect and remove anomalies, such as non-standard symbols and unexpected characters. This step ensured the integrity and quality of the text data, minimizing any potential interference during the training phase.

**Normalization:** The dialogue texts underwent normalization by removing unnecessary tabs, new lines, and carriage returns, thereby maintaining a consistent format throughout. Dialogue roles (e.g., “Patient” and “Doctor”) were identified, corrected for inconsistencies, and standardized to facilitate accurate mapping and analysis of the doctor-patient interactions.

**Dataset Conversion:** Finally, the preprocessed dialogues were converted into a JSON format suitable for model training. Each conversation was structured to include a base system prompt, followed by individual messages from the patient and doctor. This conversion prepared the dataset for fine-tuning a variant of the GPT model, allowing it to effectively understand and replicate medical dialogues.

## Enhancing GPT for healthcare interactions

Fine-tuning a pre-trained language model for a specific domain is crucial for enhancing its contextual relevance, accuracy, and overall interaction quality. In this study, the primary goal of fine-tuning was to refine the chatbot’s contextual understanding, natural language generation, and the incorporation of medical domain knowledge.

To achieve this, the MTS-Dialog dataset was employed to train the model on realistic medical dialogues Abachaa [60]. This dataset provided the necessary domain-specific information to address several key challenges, such as accurately understanding medical contexts, generating coherent and fluent responses, and ensuring precise use of medical terminology. This targeted approach ensured the chatbot could generate reliable and contextually appropriate responses tailored to healthcare interactions.

The fine-tuning process consisted of data preprocessing, model training, and evaluation. We selected the gpt-3.5-turbo-0125 model for fine-tuning due to its extensive training capacity and context length of 16,385 tokens, which were well-suited to the study’s requirements. Although newer models with longer context capabilities became available during development, the decision to proceed with gpt-3.5-turbo-0125 ensured stability and alignment with the study timeline. The training configuration involved 1,506,135 training tokens, 5 epochs, a batch size of 16, and a learning rate multiplier of 2.

In addition to these configuration choices, we also set several important model parameters. The temperature was set to 1.0, which balances randomness and coherence in responses. Top-k was set to 0, meaning it was disabled by default, as this allows the model to consider all tokens without limiting itself to the most likely ones. We used a top-p value of 1.0 for nucleus sampling, allowing the model to evaluate all probable tokens, thus enhancing the diversity of the generated responses. The maximum tokens were capped at 4096, which varies by model, to determine the maximum length of the output generated by the model. The repetition penalty was set to 1.0, meaning no extra penalty was applied for repeated tokens. Additionally, the presence penalty and frequency penalty were both set to 0.0, meaning there was no discouragement for introducing new topics or for frequently occurring tokens.

These parameters were carefully chosen to balance effective learning, maintain model stability, and mitigate overfitting, achieving optimal model generalization.

**Fig. 2:**
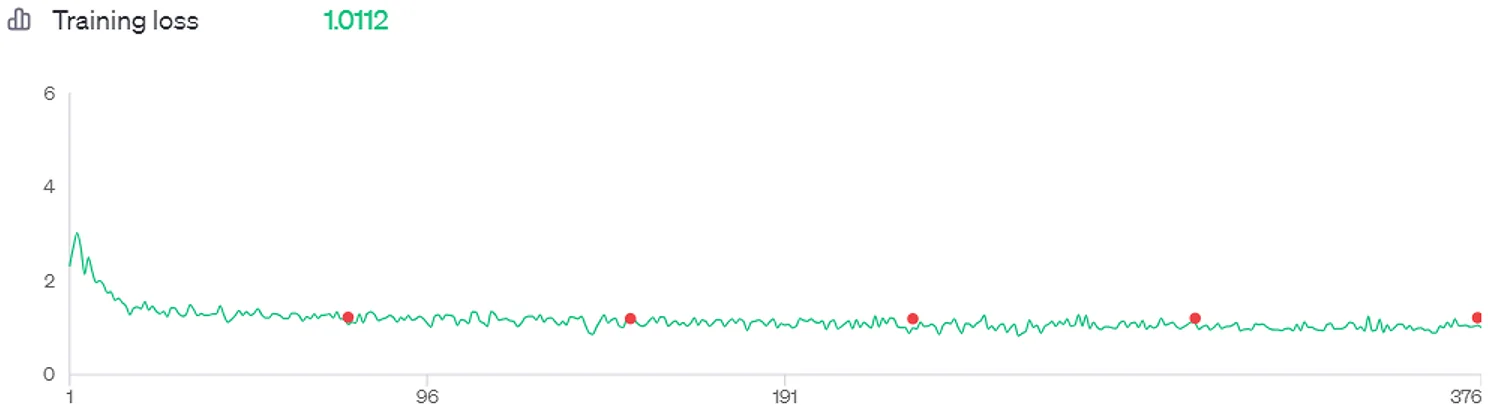
Training loss curve

The model’s learning progress was tracked through a training loss curve, depicted in Fig. 2. Initially, the training loss was high, reflecting the model’s adjustment to domain-specific medical data. As training progressed, the loss consistently declined, stabilizing at a final value of 1.0112, indicating satisfactory convergence. This reduction in training loss suggests improved accuracy and effective adaptation to the healthcare domain, highlighting the successful integration of domain-specific knowledge.

To rigorously evaluate the fine-tuned model, we used specific test sets derived from the MTS-Dialog dataset, designed to assess both the chatbot’s conversational abilities and its summarization skills. The **MTS-Dialog-TestSet-1-MEDIQA-Chat-2023** was used to evaluate the chatbot’s ability to engage in meaningful, contextually appropriate medical dialogues, while the **MTS-Dialog-TestSet-2-MEDIQA-Sum-2023** was utilized to assess its ability to generate concise and accurate summaries of medical interactions. These evaluations were critical in validating the chatbot’s readiness for real-world healthcare applications.

### Human evaluation of conversational quality

To evaluate the performance of our fine-tuned medical chatbot, we implemented a rigorous human evaluation framework. This process involved simulating realistic conversations, using GPT-4 to generate user messages and the fine-tuned model to provide responses. The primary aim of this evaluation was to assess the chatbot’s effectiveness in managing dynamic, contextually evolving interactions, which are critical in healthcare settings.

The chatbot’s outputs were subsequently reviewed by domain experts to validate the accuracy, coherence, and contextual relevance of responses. Experts rated each response based on its alignment with established medical guidelines, ensuring that the chatbot adhered to high-quality communication standards.

Furthermore, to ensure the generalizability of our findings, the evaluation dataset included a diverse range of patient scenarios, incorporating different demographics, clinical contexts, and varying complexities of medical inquiries.

#### Evaluation methodology

The human evaluation methodology employed a unique approach that simulated realistic doctor-patient interactions. For each entry in the original dataset, the initial system content was extracted, and a new conversation history was created. The interaction was initiated by the assistant, which responded to the provided content, followed by a dynamic interaction loop between the simulated patient (using GPT-4) and the assistant. This interaction spanned six conversational turns, ensuring sufficient depth and complexity to capture the nuances of healthcare dialogues.

**Fig. 3:**
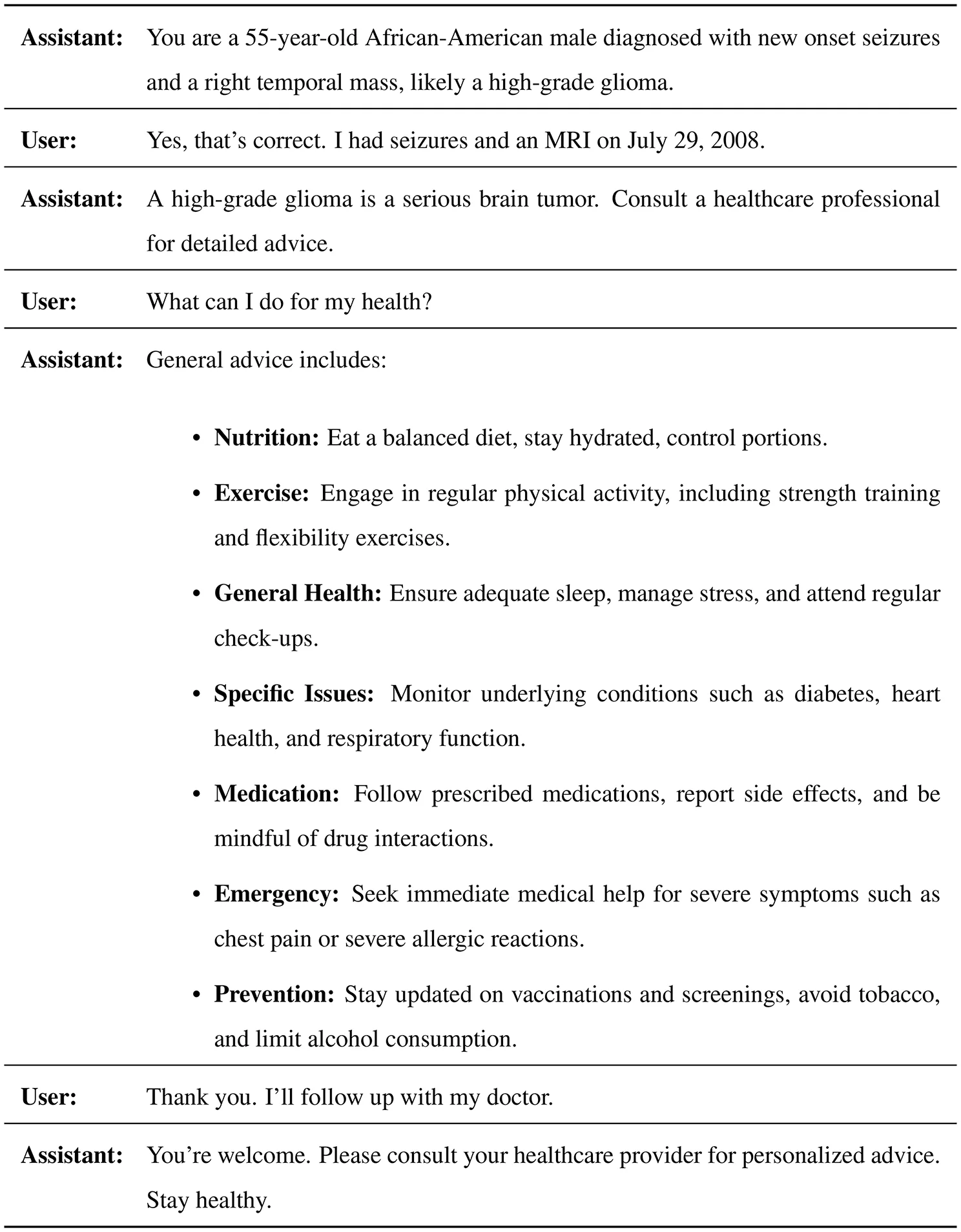
Example of a conversation between the fine-tuned model and a simulated patient (GPT-4)

This setup aimed to provide a realistic and fluid conversational experience, thereby offering a more comprehensive assessment of the model’s capability to handle context-rich and evolving medical discussions. An example of such a conversation is presented in Fig. 3, which demonstrates the chatbot’s ability to address patient inquiries effectively.

#### Evaluation criteria

To quantify the quality of the conversations generated by the model, we defined five key evaluation parameters, rated by 100 human evaluators on a scale from 0 to 5. We carefully selected the 100 medical experts from diverse healthcare backgrounds, including general practitioners, specialists, medical researchers, and health- care technology professionals. This diversity in expertise ensured a well-rounded evaluation of the chatbot’s performance across different domains within the medical field. The parameters were defined as follows:

1. **Comprehensiveness**: The extent to which the chatbot’s responses are thorough, providing detailed information that effectively addresses the user’s concerns.
2. **Engagement**: The chatbot’s ability to maintain the user’s interest and encourage continued interaction throughout the conversation.
3. **Empathy and Tone**: The chatbot’s success in conveying empathy, ensuring the tone is appropriate for sensitive medical contexts.
4. **Clarity**: The clarity and understandability of the chatbot’s responses, free from ambiguity and accurately conveying the intended message.
5. **Language Interactiveness**: The chatbot’s capacity to foster a dynamic and engaging two-way conversation, enhancing user experience.

These criteria were not adopted from standard evaluation metrics; rather, we designed our own after surveying the existing literature and consulting with experts in the field to ensure a comprehensive assessment of the chatbot’s capabilities. The criteria focus on both the technical accuracy and the quality of human interaction, which is vital in healthcare communication. Our approach aimed to fill gaps identified in previous evaluation frameworks, which often overlooked aspects such as empathy, engagement, and the nuanced quality of conversation crucial for medical interactions.

#### Results and analysis

The evaluation scores assigned by the human annotators were aggregated to determine the average performance across each parameter. A detailed summary of these scores is presented in Table 4. To assess the consistency among evaluators, Krippendorff’s Alpha (*α*) was calculated, yielding a value of 0.81, indicating substantial agreement among the evaluators. This high level of consensus underscores the reliability of the evaluation process and supports the validity of the findings.

**Table 4:** Average review score for human evaluators

Aspect	Average Score (0–5)
Comprehensiveness	4.57
Engagement	4.49
Empathy and Tone	4.53
Clarity	4.68
Language Interactiveness	4.23
**Krippendorff’s Alpha**	**0.81**

Krippendorff’s Alpha is a statistical measure used to evaluate the level of agreement between multiple raters. It is suitable for different types of data, such as nominal, ordinal, interval, and ratio scales. The formula for calculating Krippendorff’s Alpha is:

1 $$\alpha = 1 - \frac{D_o}{D_e}$$

Where:

- $D_o$ is the observed disagreement, which measures the extent to which the ratings by different evaluators deviate from one another.
- $D_e$ is the expected disagreement, which represents the disagreement that would be expected by chance.

When *α* is close to 1, it indicates a high level of agreement among raters, while values closer to 0 suggest low agreement. The obtained value of 0.81 indicates substantial agreement, which, according to established guidelines, is considered a reliable level of consistency among raters. This strengthens the credibility of the evaluations used in our study, suggesting that the scoring system effectively captured the evaluative criteria across different raters with minimal subjective bias.

The substantial agreement reflected by Krippendorff’s Alpha validates the robustness of the evaluation process, ensuring that the insights derived from these assessments are both reliable and meaningful. The relatively high scores across all parameters indicate that the fine-tuned model effectively meets the core requirements of healthcare interactions, including maintaining clarity, demonstrating empathy, and engaging users meaningfully.

## Empowering GPT as an AI medical agent

Transforming a general-purpose language model like GPT into an effective AI medical agent requires the integration of various advanced tools and technologies to enable accurate, context-aware, and reliable healthcare interactions. Our approach leverages RAG for accessing hospital reviews, the Wikipedia API for up-to-date information retrieval, and a knowledge graph for patient symptoms and diseases. These components collectively empower the model to access a vast repository of medical knowledge, ensuring the generation of precise and contextually appropriate medical advice.

AI medical agents have the potential to transform healthcare by supporting healthcare professionals and enhancing patient engagement. They can provide rapid access to medical information, assist in diagnostic processes, and deliver treatment recommendations. The integration of these AI systems into healthcare not only enhances decision-making efficiency and accuracy but also helps reduce the workload of healthcare professionals, ultimately contributing to better patient outcomes and quality of care.

### Retrieval-Augmented generation for hospital reviews

Retrieval-Augmented Generation is an innovative technique that enhances language models by combining the strengths of information retrieval and natural language generation. The primary benefit of RAG lies in its ability to retrieve relevant information from a large corpus and use that context to generate accurate and informed responses. Traditional language models, despite their capacity to process extensive datasets, often face limitations regarding outdated or incomplete information and challenges in accessing real-world knowledge. These limitations can result in inaccuracies, which are especially critical in medical contexts.

To address these challenges, we implemented RAG using a dataset comprising hospital reviews. By retrieving contextually relevant information from this dataset, the model can generate responses informed by real patient experiences, thereby enhancing the quality and accuracy of its advice. This integration ensures that users receive comprehensive and reliable information, leading to more informed healthcare decisions.

**Fig. 4:**
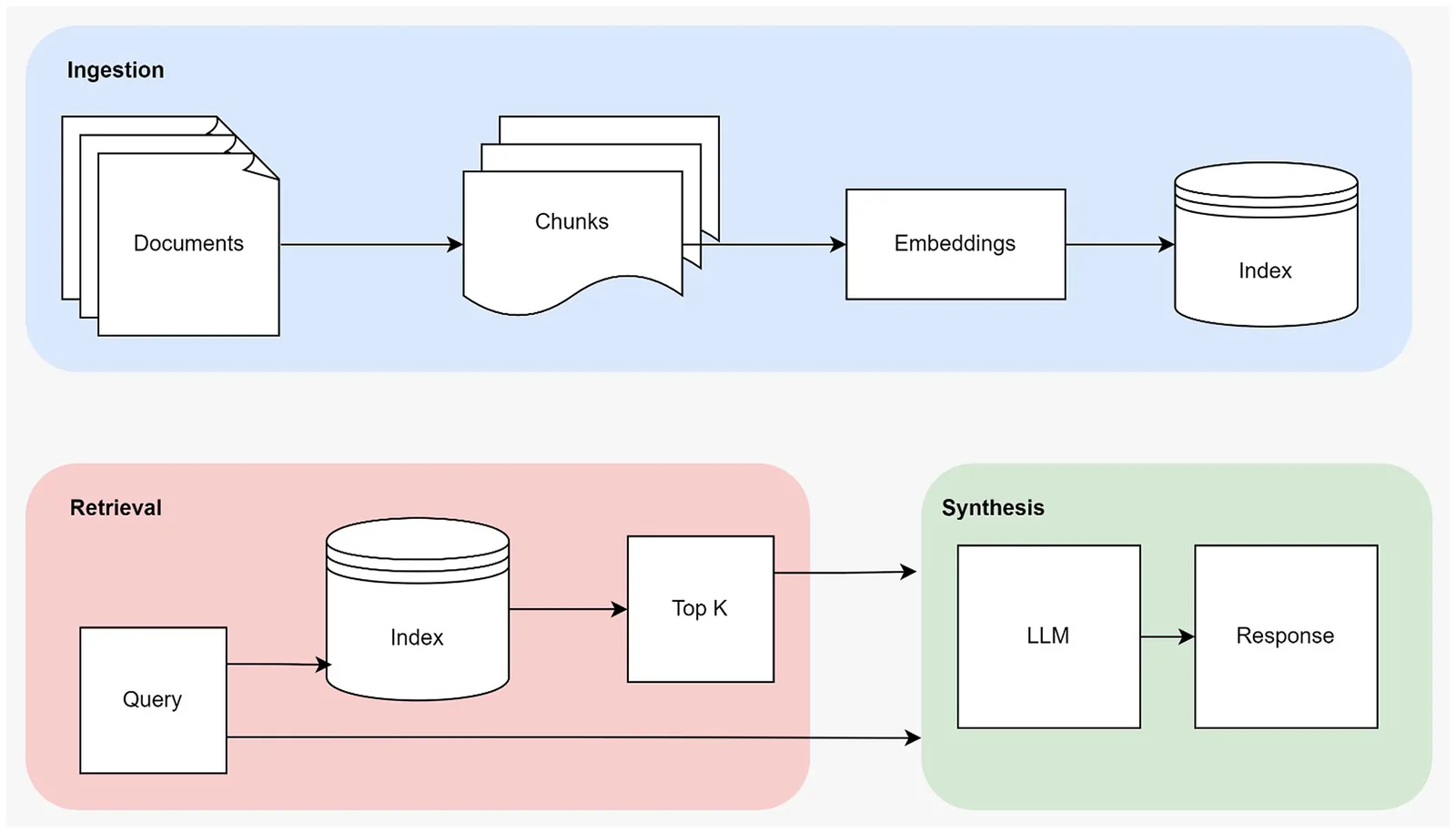
Overview of the retrieval-Augmented generation (RAG) process

The RAG workflow involves three main stages: ingestion, retrieval, and synthesis, as illustrated in Fig. 4. During the ingestion phase, documents are split into smaller chunks, converted into vector embeddings, and stored in an index. The retrieval phase involves searching the index based on a user query to identify the most relevant chunks. Finally, during the synthesis stage, the selected chunks are used by the language model to generate a coherent response, ensuring the output is both informative and contextually appropriate.

#### Document processing and embedding generation

In our implementation, each row of the hospital reviews dataset represents an independent review, which is preserved as a distinct chunk to maintain its context. Document chunking is thus simplified, as no predefined function is needed to split the data. To convert these chunks into vector embeddings, we employed the text-embedding-ada-002 model, known for its robustness in capturing the semantic meaning of text Nussbaum et al. [63]. These embeddings allow the chatbot to perform similarity searches and retrieve the most relevant content to answer user queries accurately. The model is cost-effective, with a pricing structure of $0.100 per 1,000,000 tokens, suitable for processing large volumes of text OpenAI [64].

#### Vector storage and retrieval

The generated embeddings are stored in Chroma, an open-source vector database selected for its scalability and seamless integration capabilities. Each embedding is stored along with metadata, including document ID and chunk position, enabling efficient retrieval. The use of Chroma ensures rapid access to the most relevant reviews based on their semantic content, thereby enhancing the chatbot’s response accuracy and depth of information Chroma [65].

### Wikipedia API integration

To provide detailed and up-to-date information on medical topics, we integrated the Wikipedia API into our system. This allows the model to dynamically access a broad and evolving source of general medical knowledge, improving its ability to handle a wide range of queries.

However, recognizing that Wikipedia content may vary in reliability, we apply a basic filtering mechanism to ensure the credibility of retrieved information. Specifically, we favor articles with a high number of citations, prioritize pages labeled as *Good Article* or *Featured Article*, and exclude entries flagged with warning tags such as “multiple issues” or “needs additional citations.” Preference is also given to well-referenced sections citing reputable external sources.

Furthermore, all retrieved Wikipedia information is cross-validated against validated clinical guidelines and peer-reviewed medical sources. In cases of conflict, the system prioritizes the knowledge graph content over Wikipedia-derived information.

Wikipedia content is therefore used as a supplementary source to enrich user interactions, without serving as the sole basis for clinical decision-making. This design ensures that the system maintains high standards of accuracy, reliability, and safety in medical information delivery.

### Implementing a symptom checker tool

To further augment user interaction, we implemented a symptom checker tool using GPT-4 and a customized prompt template. This tool allows users to input their symptoms and receive information on potential conditions and recommended actions. By leveraging the model’s extensive knowledge base and reasoning capabilities, the symptom checker provides users with relevant and timely health information, empowering them to make informed decisions about their health.

### Knowledge graph for patient symptoms and diseases

**Fig. 5:**
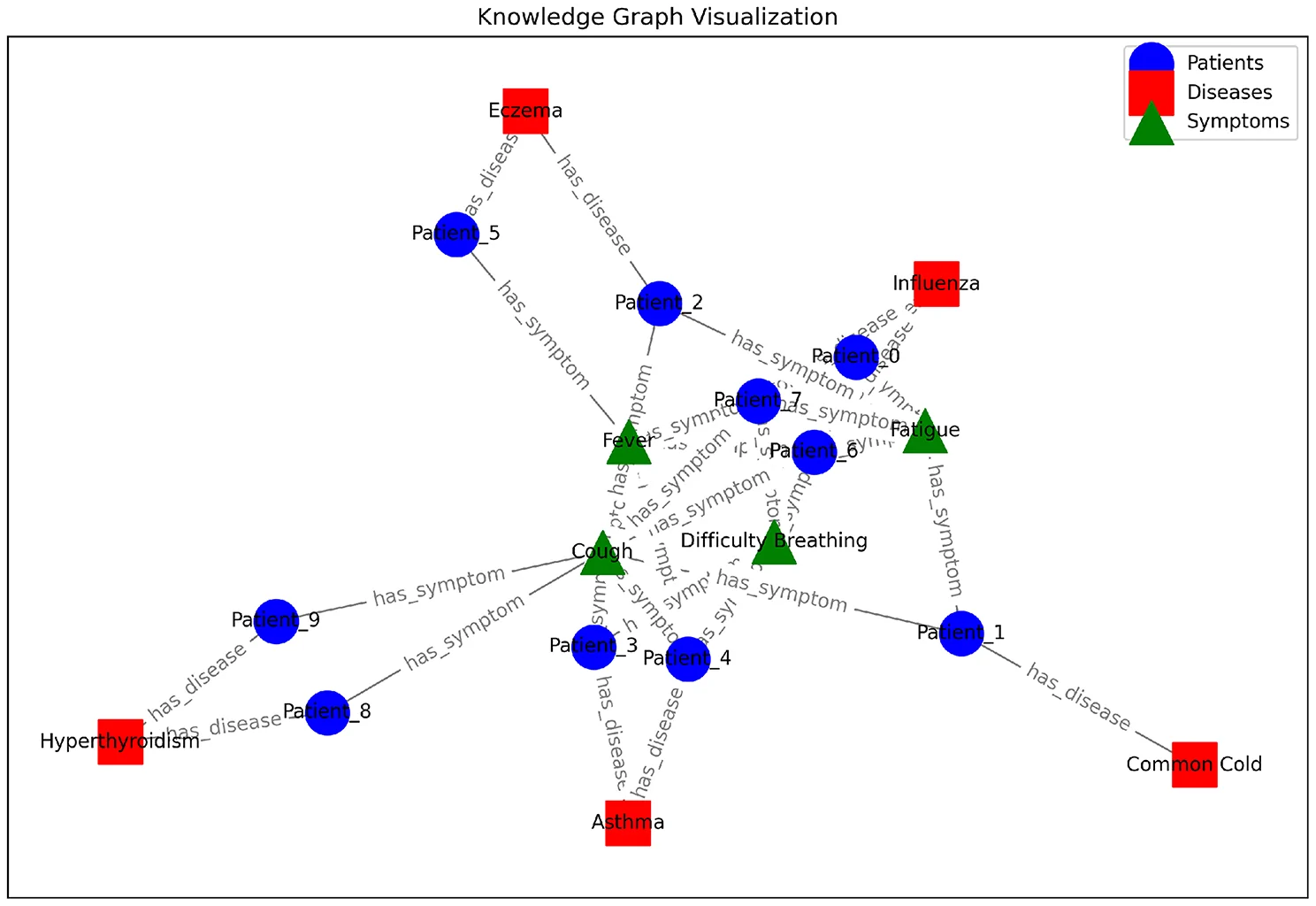
Knowledge graph representation of medical data

A knowledge graph was implemented to represent and query complex relationships within medical data, thereby providing a structured view of patients, diseases, and symptoms. As shown in Fig. 5, the knowledge graph allows efficient and precise information retrieval, facilitating detailed insights into patient profiles and their associated conditions.

The construction of the knowledge graph involved several steps:

1. **Data Loading:** Patient data, diseases, and symptoms were sourced from a comprehensive CSV file and loaded using the Pandas library for efficient manipulation.
2. **Graph Initialization:** An empty graph was initialized using the **NetworkX** library, which provides robust tools for creating and managing complex networks.
3. **Node and Edge Addition:** Nodes were added for each patient, disease, and symptom, with edges denoting relationships such as has_disease and has_symptom. This structure ensures that each patient is accurately represented within the graph, enabling effective queries.

#### Querying the knowledge graph

**Fig. 6:**
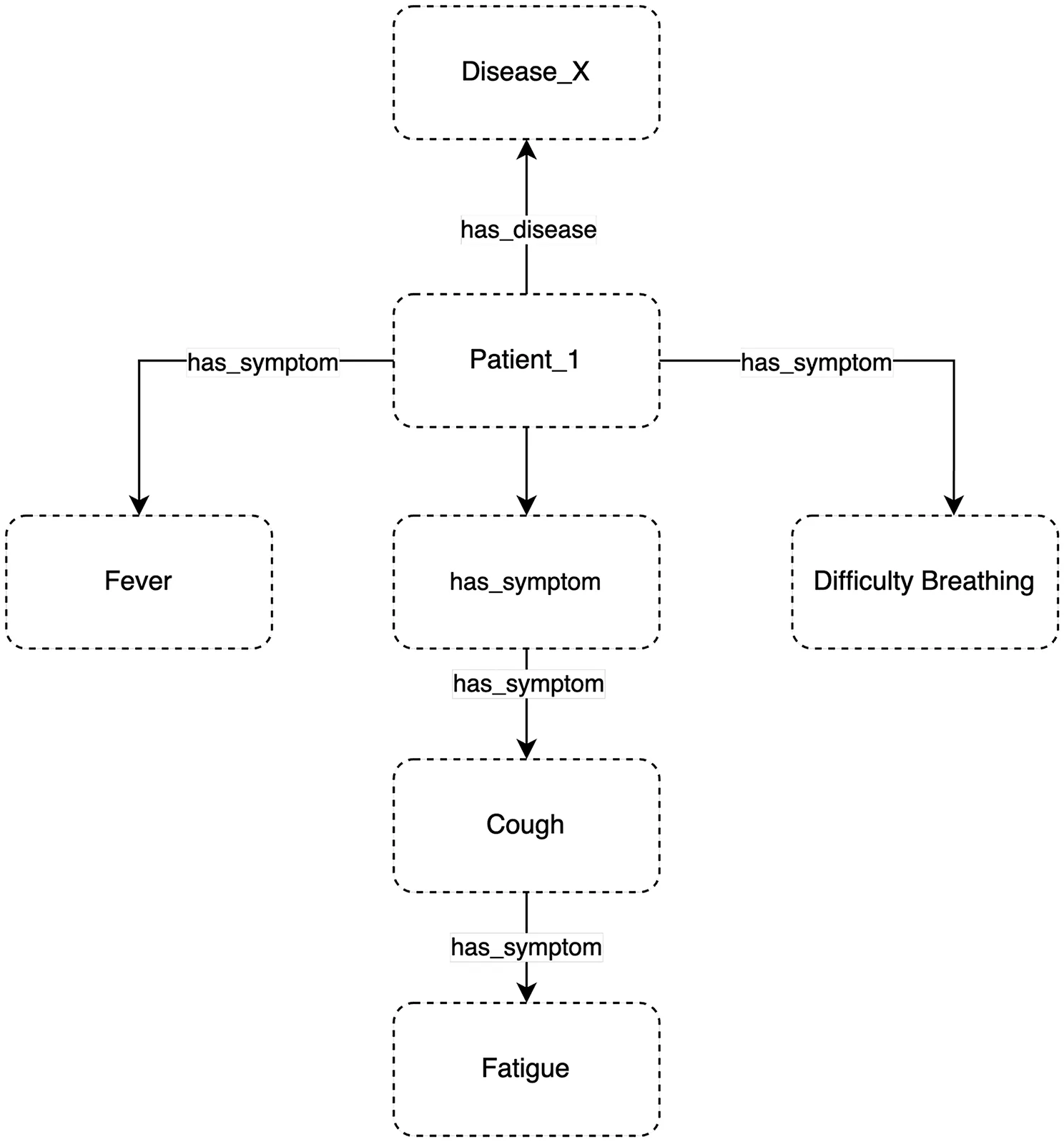
Querying the knowledge graph for patient, disease, and symptom relationships

As shown in Fig. 6, the knowledge graph supports efficient querying, allowing the extraction of detailed information about patients, symptoms, and diseases. Queries can be categorized into:

- **Patient-Centric Queries:** Retrieve all diseases and symptoms associated with a specific patient.
- **Disease-Centric Queries:** Identify symptoms related to a specific disease by tracing connections from the disease node.
- **Symptom-Centric Queries:** Find all patients exhibiting a particular symptom by locating the relevant symptom node.

#### Natural language query processing

To enhance user interaction with the knowledge graph, an NLP function was developed to convert user queries into graph queries. This function interprets user inputs to determine the type of entity (e.g., patient, disease, symptom) and generates a structured query for the knowledge graph. This ensures a user-friendly interface, allowing seamless access to the information contained within the graph.

For example:

- If a user asks, “What are the symptoms of diabetes?” the NLP function identifies the entity type as a “disease” and generates a graph query to retrieve all nodes and edges related to “diabetes,” specifically extracting the associated symptoms.
- If a user queries, “Which patients have reported experiencing fatigue?” the function identifies “fatigue” as a symptom and forms a query that retrieves all patients linked to the “fatigue” node in the knowledge graph.
- Another example could be, “What treatments are available for patient John Doe?” where the NLP function identifies “John Doe” as a patient entity and constructs a query to find all treatment nodes connected to this specific patient.

These examples illustrate how the NLP function translates natural language queries into specific graph queries, enabling users to interact effectively with complex medical data without needing any knowledge of query syntax. This approach ensures an intuitive experience, allowing users to seamlessly retrieve relevant and structured medical information.

## Comprehensive system architecture

**Fig. 7:**
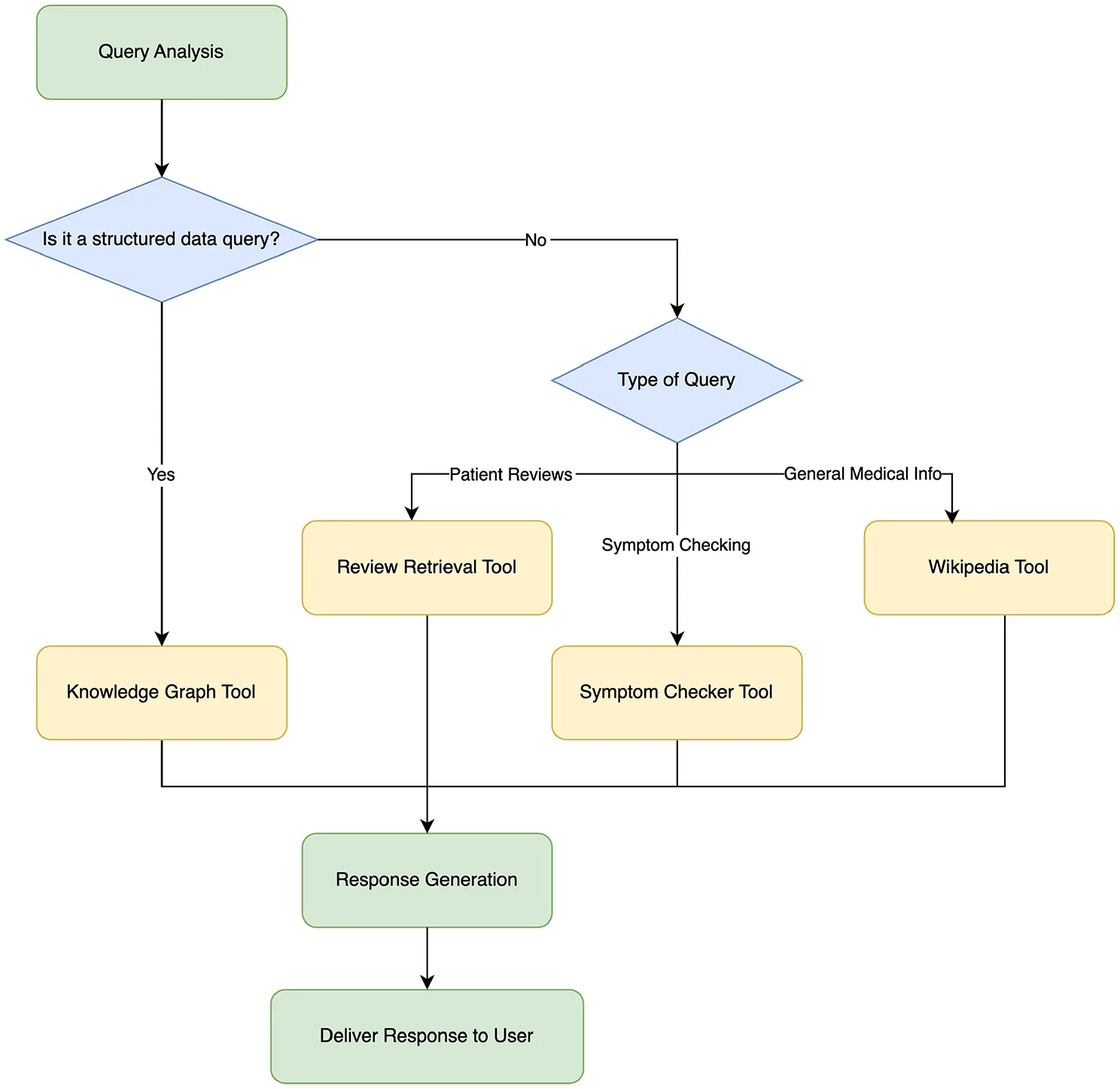
Overview of the system architecture

The system architecture, as shown in Fig. 7, is designed to efficiently manage medical information retrieval, symptom checking, and user query handling. It employs a nested structure that integrates multiple advanced AI tools, ensuring accurate, context-aware responses. The key components of this architecture include GPT-4 and a fine-tuned version of GPT-3.5, a knowledge graph derived from medical datasets, Wikipedia API integration, a Chroma-based vector database for patient reviews, and a dynamic query management agent.

### Dynamic query management

The system’s query management is orchestrated by an intelligent agent that dynamically selects the appropriate tool for each query. The agent employs a zero-shot learning approach, enabling it to generalize to new, unseen queries. This approach allows the agent to determine the context and requirements of each query effectively and then select the optimal tool, such as the knowledge graph, symptom checker, or Wikipedia integration.

The dynamic selection process ensures that each query is processed by the most suitable tool, optimizing the relevance and accuracy of the responses. For example, a query about patient symptoms triggers the symptom checker tool, while a general medical question may utilize the Wikipedia integration. This flexibility allows for a seamless and context-aware interaction.

The system also includes robust error-handling mechanisms to address a wide range of user queries and potential parsing issues:

- Error Detection and Correction: Identifies and corrects common parsing errors, ensuring queries are accurately processed and minimizing the risk of incomplete or incorrect responses.
- Fallback Strategies: Provides general responses or prompts users for clarification if a query cannot be fully handled. This ensures the user receives assistance even for more challenging questions.
- Contextual Awareness: Maintains a consistent understanding of the interaction, allowing for coherent responses to follow-up questions and enhancing the overall user experience.

### System workflow

The overall system workflow is designed to process user queries in three main stages: Query Analysis, Tool Selection, and Response Generation.

#### Query analysis

The initial stage involves analyzing the user’s query to determine its intent and requirements. Sophisticated natural language processing techniques are employed to extract key entities, keywords, and contextual indicators. This analysis helps classify the query and decide if it requires structured data retrieval (e.g., patient-specific information) or general information.

#### Tool selection

Once the query has been analyzed, the system dynamically selects the most appropriate tool to generate a response:

- Knowledge Graph: Invoked for queries that involve specific medical entities and relationships. It provides structured and relational data, making it ideal for questions about patient symptoms, diseases, or treatment options.
- Wikipedia Integration: Selected for general informational queries, leveraging Wikipedia’s extensive database to provide detailed, up-to-date medical knowledge.
- Symptom Checker Tool: Utilized for queries involving user symptoms, generating personalized advice based on potential medical conditions.
- Review Retrieval Tool: Engages the vector database to provide insights based on real patient reviews, adding contextual value to queries about hospital experiences or treatment feedback.

This dynamic selection mechanism ensures that the tool used is the best fit for the type of information requested, thus enhancing the relevance and accuracy of the responses.

#### Response generation

The final stage involves generating a response tailored to the user’s needs based on the selected tool:

- Knowledge Graph Queries: Extract and present structured information about medical entities and their relationships, ensuring accuracy and clarity in responses.
- Wikipedia and Symptom Checker Queries: Utilize GPT-4 to generate detailed and contextual responses. The Wikipedia tool provides broad medical explanations, while the symptom checker interprets user inputs to deliver personalized diagnostic information.
- Review Retrieval Queries: The Chroma vector database is accessed to synthesize responses based on patient feedback, offering insights grounded in real-world experiences.

The system maintains contextual awareness throughout the interaction to manage follow-up questions, thereby providing a coherent and user-friendly experience.

### Incorporating historical context

To ensure contextual relevance and coherence, the system incorporates a historical context mechanism that accounts for previous user interactions. Each new query is appended with relevant past interactions, enabling the system to generate responses informed by the entire conversation history. This approach enhances the quality of the dialogue, maintaining continuity and delivering contextually aware responses, ultimately improving the user experience.

### Comprehensive integration for medical assistance

By integrating advanced language models, a knowledge graph, Wikipedia API, a symptom checker, and a vector database, this architecture provides a robust and comprehensive solution for medical information retrieval. The dynamic query handling agent, combined with sophisticated error management and contextual awareness, ensures that users receive precise, reliable, and contextually appropriate information. This multi-faceted approach not only enhances the accuracy of medical advice but also significantly improves patient engagement and healthcare support.

## Results and discussion

The system architecture incorporates multiple tools, each designed to provide distinct capabilities for retrieving and synthesizing medical information. The effectiveness of these tools was evaluated through a series of test scenarios, demonstrating their ability to deliver relevant, accurate, and contextually informed responses. To further validate the approach, we introduce quantitative metrics such as accuracy, response time, and user satisfaction scores, complemented by appropriate visualizations.

### Knowledge graph utilization

To comprehensively assess the effectiveness of the knowledge graph, additional analysis was conducted across multiple experimental scenarios. These experiments aimed to demonstrate the system’s capability to manage diverse and complex medical queries efficiently. The scenarios tested included:

- **Symptom-based Querying:** Retrieving relevant symptoms associated with a particular disease.
- **Patient Identification:** Identifying patients exhibiting specific symptoms or conditions.
- **Profile-based Information Extraction:** Extracting detailed medical histories or treatment records from individual patient profiles.

To quantitatively measure its accuracy, the system was evaluated using a predefined test set (around 10% of the original dataset) containing ground-truth relationships between medical entities (e.g., diseases, symptoms, and treatments). The knowledge graph achieved an impressive **95% accuracy**, highlighting its **high reliability in retrieving precise and contextually relevant medical information** for decision-making processes.

**Fig. 8:**
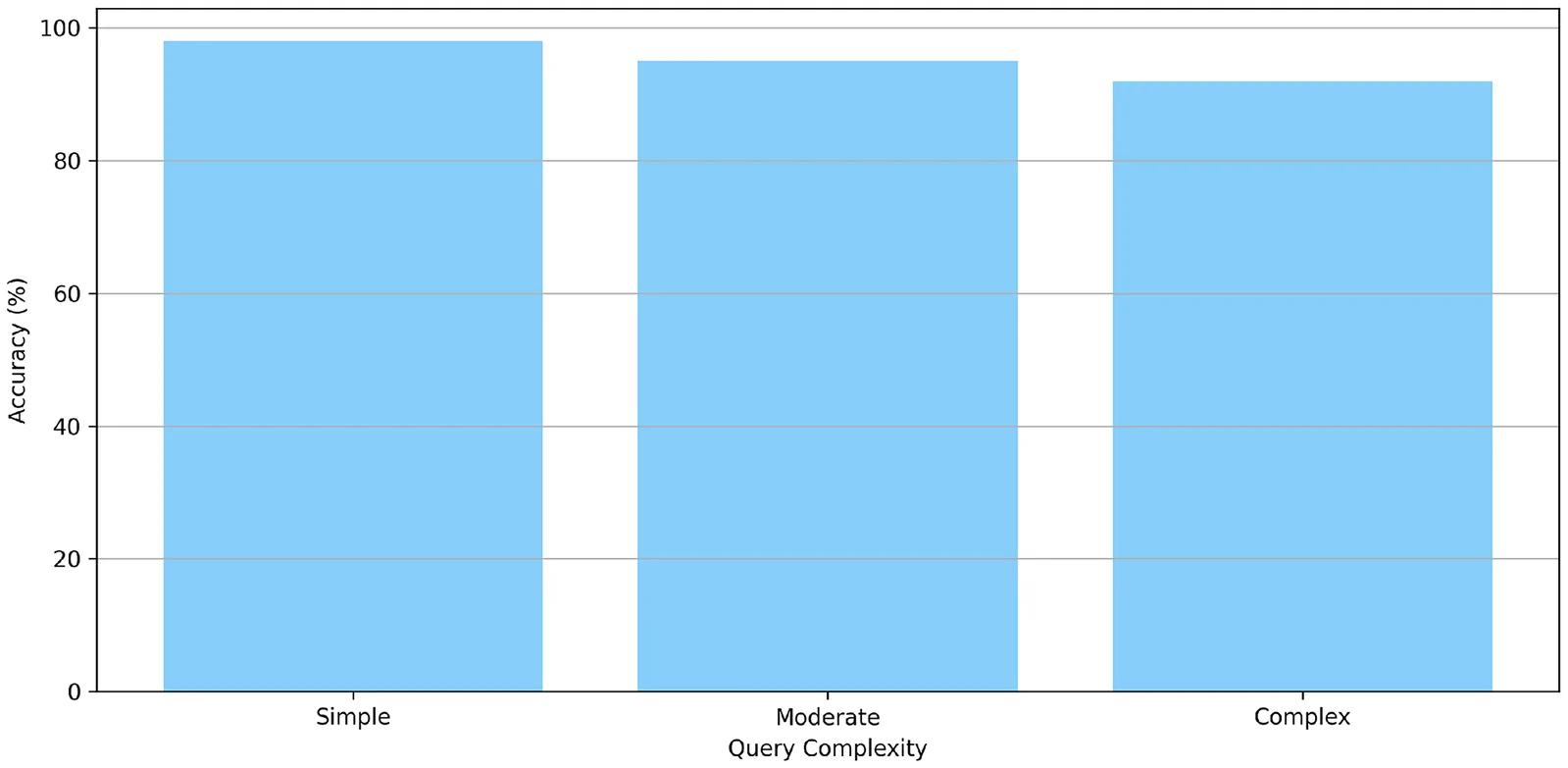
Knowledge graph accuracy vs. Query complexity

To further analyze the system’s robustness, its performance was examined across queries of varying complexity levels: simple, moderate, and complex. As illustrated in Fig. 8, the system maintained consistently high accuracy across all levels, demonstrating its adaptability in handling different types of queries.

- For **simple queries**, where direct relationships between entities were queried (e.g., *“What are the symptoms of influenza?”*), the accuracy reached **98%**.
- For **moderate queries**, which involved intermediate-level reasoning (e.g., *“Which patients exhibit symptoms of both diabetes and hypertension?”*), the accuracy slightly declined but remained strong at **96%**.
- For **complex queries**, requiring deeper reasoning and multiple relational hops (e.g., *“Which treatments are most commonly prescribed for patients exhibiting symptoms X, Y, and Z, considering their medical history?”*), accuracy marginally decreased to **92%**.

These results emphasize the **scalability and robustness** of the knowledge graph, ensuring reliable performance even for intricate medical queries. The minimal drop in accuracy as query complexity increased suggests that the system is well-equipped for real-world medical information retrieval, where queries often involve multiple interrelated factors.

By systematically validating the knowledge graph across different use cases and levels of complexity, these findings reinforce its **potential for assisting in medical research, diagnostics, and personalized treatment recommendations**.

Several test cases provide further evidence of the knowledge graph’s capabilities in processing complex queries and delivering precise, structured responses. These examples are critical for supporting accurate medical decision-making:

- **Scenario 1: Querying for Symptoms of a Disease**
  - **Input:** “What are the symptoms of eczema?”
  - **Output:** “Symptoms: Fever, Cough, Fatigue.”
  This scenario illustrates the graph’s ability to quickly retrieve information on disease symptoms, providing clear and actionable responses.
- **Scenario 2: Querying for Patients with a Specific Symptom**
  - **Input:** “Which patients have the symptom fever?”
  - **Output:** “Patient: Patient_0, Patient_3, Patient_4, Patient_5, Patient_6, … “
  In this case, the knowledge graph successfully identified all patients exhibiting the symptom “fever,” showcasing its ability to process more intricate queries involving patient profiles.
- **Scenario 3: Querying for Symptoms and Diseases of a Patient**
  - **Input:** “What are the symptoms of patient 11?”
  - **Output:** “Disease: Allergic Rhinitis; Symptoms: Cough, Fatigue.”

This example demonstrates the system’s capability to retrieve detailed patient-specific information, including both symptoms and associated diseases, ensuring that medical professionals can access the comprehensive data required for patient care.

To assess the accuracy of the knowledge graph tool in mapping symptoms to diseases, we evaluated its predictions using a reserved validation dataset consisting of diverse medical cases. This evaluation aimed to measure the tool’s ability to generate correct associations while identifying areas where misclassifications occurred.

The knowledge graph successfully identified the expected disease when provided with relevant symptoms. Below are examples demonstrating its correct performance:

- **Case 1:**
  - **Input Symptoms:** Persistent thirst, frequent urination, unexplained weight loss
  - **Expected Disease:** Diabetes Mellitus
  - **Predicted Disease:** Diabetes Mellitus (Correct)
  - **Explanation:** These symptoms strongly correlate with diabetes, and the tool correctly mapped them to the disease.
- **Case 2:**
  - **Input Symptoms:** Chest pain, shortness of breath, nausea
  - **Expected Disease:** Myocardial Infarction (Heart Attack)
  - **Predicted Disease:** Myocardial Infarction (Correct)
  - **Explanation:** The knowledge graph accurately retrieved this association, reflecting its ability to capture key disease markers.

In some instances, the knowledge graph failed to correctly assign the disease, primarily due to overlapping symptoms among multiple conditions. Such an example includes:

- **Case 3:**
  - **Input Symptoms:** High fever, muscle pain, rash
  - **Expected Disease:** Dengue Fever
  - **Predicted Disease:** Influenza *×* (Incorrect)
  - **Error Analysis:** While both conditions share flu-like symptoms, the model failed to recognize the presence of a rash as a distinguishing characteristic of dengue fever.

These results highlight both the strengths and limitations of the knowledge graph tool. The correctly mapped cases demonstrate its potential in assisting medical professionals, while the incorrectly mapped cases suggest areas for refinement, such as improved symptom weighting.

The performance of the knowledge graph across various complexity levels is visualized in Fig. 8. This figure highlights the tool’s ability to maintain high accuracy even as query complexity increases, affirming its value in medical decision-making processes.

### Wikipedia tool performance

**Fig. 9:**
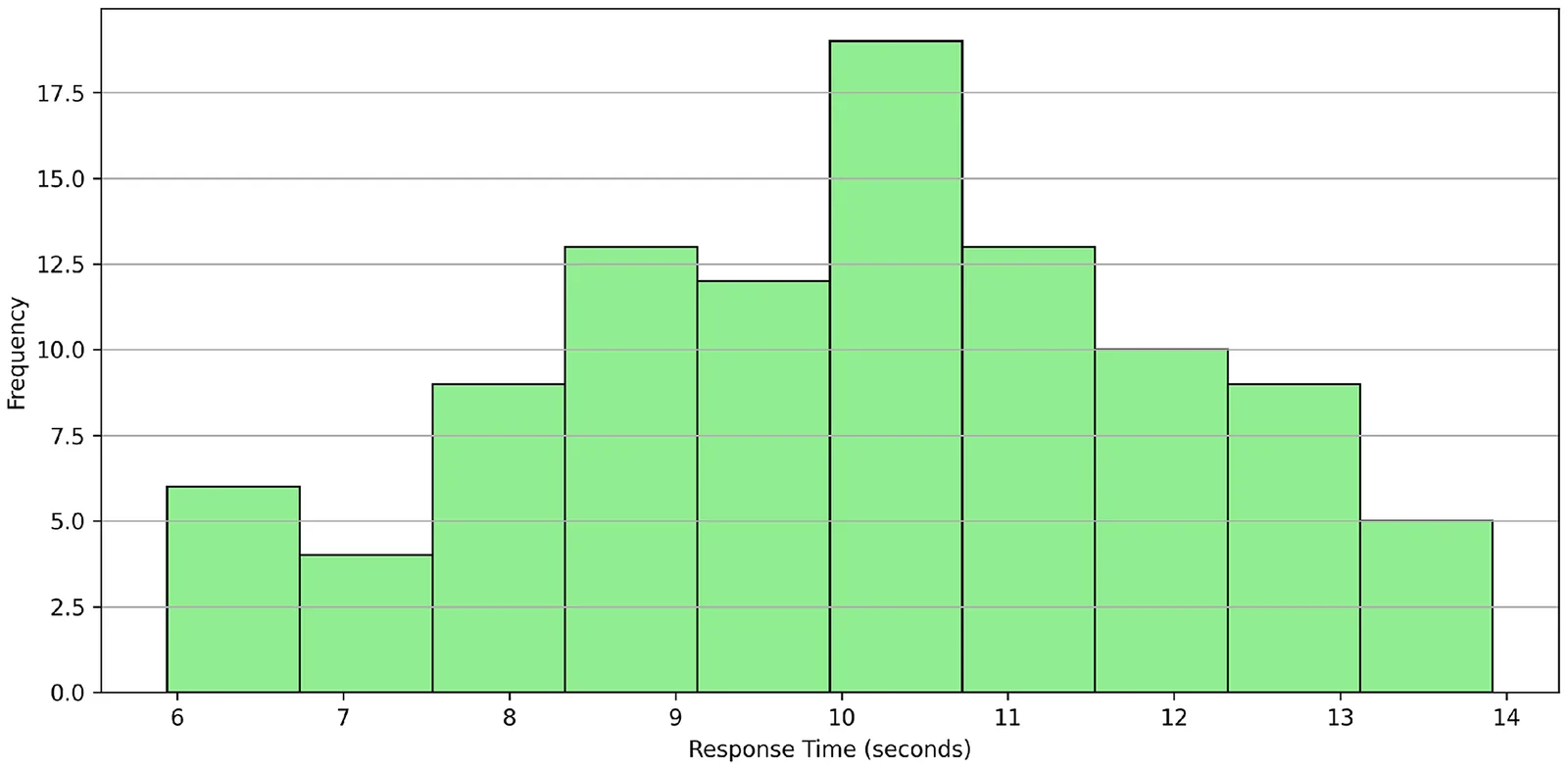
Wikipedia tool response time distribution

The Wikipedia tool was assessed for its ability to provide comprehensive and accurate explanations for medical-related queries. One of the critical performance metrics was response time, which is essential for ensuring a seamless user experience during real-time interactions. Figure 9 illustrates the distribution of response times, with an average of 10 seconds.

To improve efficiency and reduce latency, we implemented a caching system that stores previously retrieved responses for frequently asked queries. This system works by maintaining a local cache where responses are temporarily saved. When a user submits a query, the system first checks if a cached response exists. If a match is found, the cached result is instantly retrieved, significantly reducing the need for redundant requests. Otherwise, the system fetches fresh data from Wikipedia, stores it in the cache for future use, and then delivers it to the user. By leveraging this caching mechanism, the average response time was reduced to 3 seconds, ensuring a more responsive and seamless user experience while maintaining the accuracy and comprehensiveness of the retrieved information.

The primary purpose of the Wikipedia tool in this system is to serve as a reliable and dynamic knowledge source for medical queries, enhancing the breadth and depth of the AI-driven responses. The system not only retrieves relevant medical explanations but also ensures their real-time availability, addressing the challenge of keeping responses up-to-date with evolving medical knowledge. Furthermore, response time optimization plays a crucial role in improving user experience, particularly in healthcare applications where timely information is vital. The caching mechanism significantly enhances system efficiency by minimizing redundant API requests and reducing load on external resources. This improvement is especially beneficial for high-traffic scenarios where multiple users may inquire about similar topics, as it ensures rapid access to critical information without sacrificing accuracy.

In addition to response time improvements, our analysis indicates that the caching system contributes to overall system robustness. By reducing dependency on live API calls, it mitigates potential downtimes or delays caused by network fluctuations or Wikipedia API rate limits. Moreover, user engagement is expected to improve due to faster interactions, which aligns with the overarching goal of delivering a seamless, AI-powered healthcare support system.

An example scenario demonstrating the tool’s capability is shown below, where a query about diabetes treatment was handled effectively:

**Scenario: Treatment for Diabetes**

- **Input:** “Tell me about the treatment for diabetes.”
- **Output:** “The main goal of diabetes management is to restore carbohydrate metabolism to a normal state. This can be achieved through insulin replacement therapy for individuals with insulin deficiency. Lifestyle changes such as dietary modifications and exercise can also help manage symptoms. Medications like Tirzepatide are used for type 2 diabetes and can also aid in weight loss.”

This example showcases the tool’s ability to retrieve detailed and relevant information on complex medical topics, enhancing its value as a real-time reference for users.

### Symptom checker tool evaluation

**Fig. 10:**
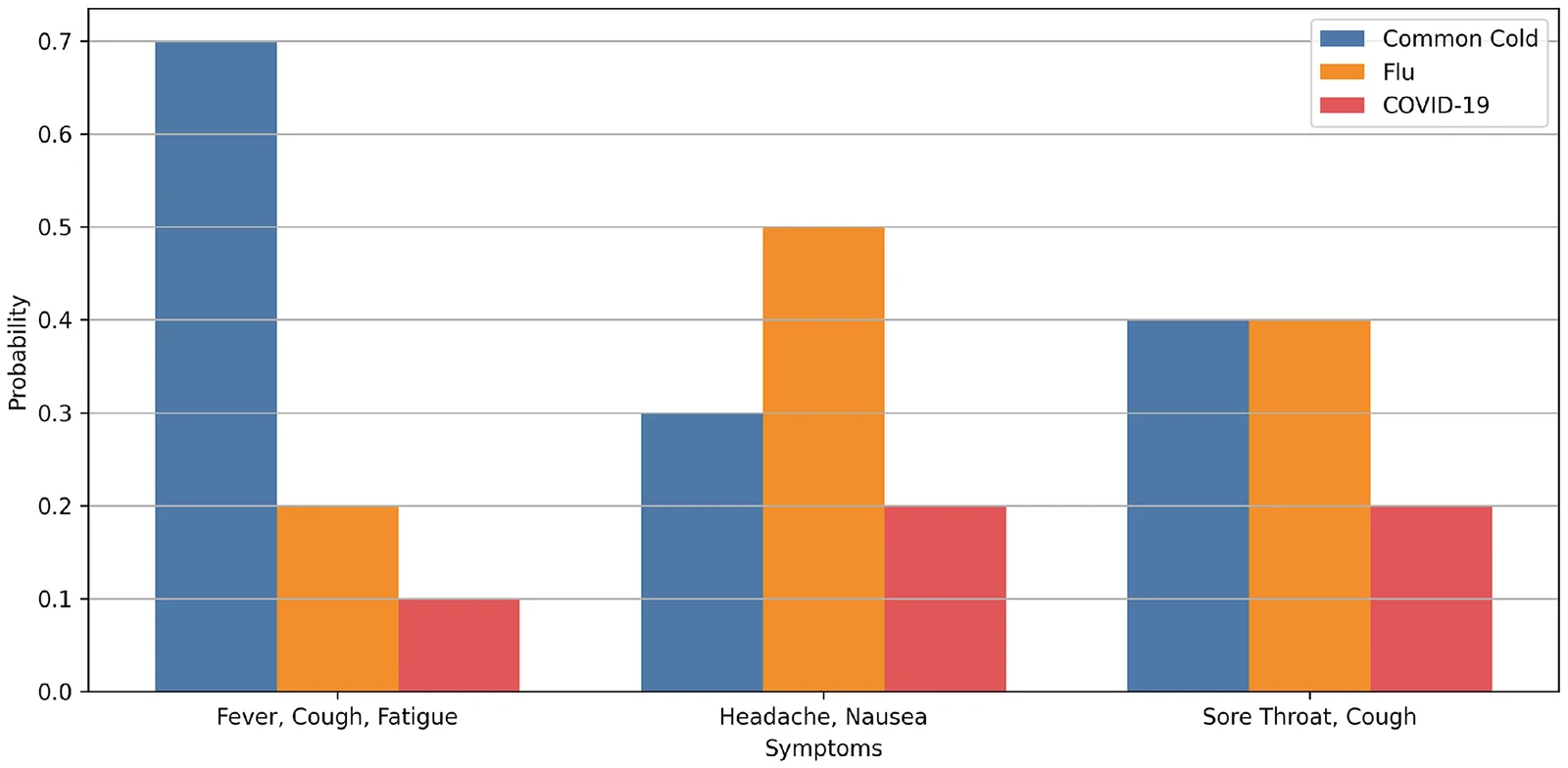
Diagnosis probability distribution for symptom checker tool

The symptom checker tool was designed to provide potential diagnoses based on the symptoms entered by the user. To evaluate its performance, the tool was tested with a variety of common symptom combinations. In addition to qualitative assessment, the tool’s ability to assign probabilities to different diagnoses, such as the common cold, flu, or COVID-19, was quantitatively measured. The results of these calculations are visualized in Fig. 10.

This plot demonstrates how the tool distributes probabilities across possible conditions for a given set of symptoms. For instance, when presented with “Fever, Cough, Fatigue,” the tool assigned a 70% probability to the common cold, 20% to the flu, and 10% to COVID-19. Such distributions highlight the tool’s ability to suggest a range of likely diagnoses, assisting users in making informed decisions about their health.

By leveraging probabilistic reasoning, the symptom checker provides a nuanced approach to preliminary diagnosis, ensuring that users are aware of potential conditions rather than offering a singular, definitive diagnosis. This method increases transparency and encourages users to seek professional medical advice for confirmation. Furthermore, response accuracy plays a vital role in user trust and engagement, and improving the algorithm’s precision ensures that the tool remains a reliable resource for health assessment.

A scenario-based example further illustrates the tool’s diagnostic capabilities:

**Scenario: Diagnosing Based on Symptoms**

- **Input:** “Symptoms: Fever, Cough, Fatigue. What could be the condition?”
- **Output:** “These symptoms could indicate a common cold, flu, or COVID-19. It is important to rest, stay hydrated, and consider over-the-counter medications. Due to the ongoing pandemic, getting tested for COVID-19 is recommended, and isolation may be necessary until test results are obtained. Seek immediate medical attention if symptoms worsen.”

This example scenario showcases the tool’s practical utility in guiding users toward potential diagnoses and recommending next steps, such as testing or seeking medical attention.

### Review retrieval tool assessment

**Fig. 11:**
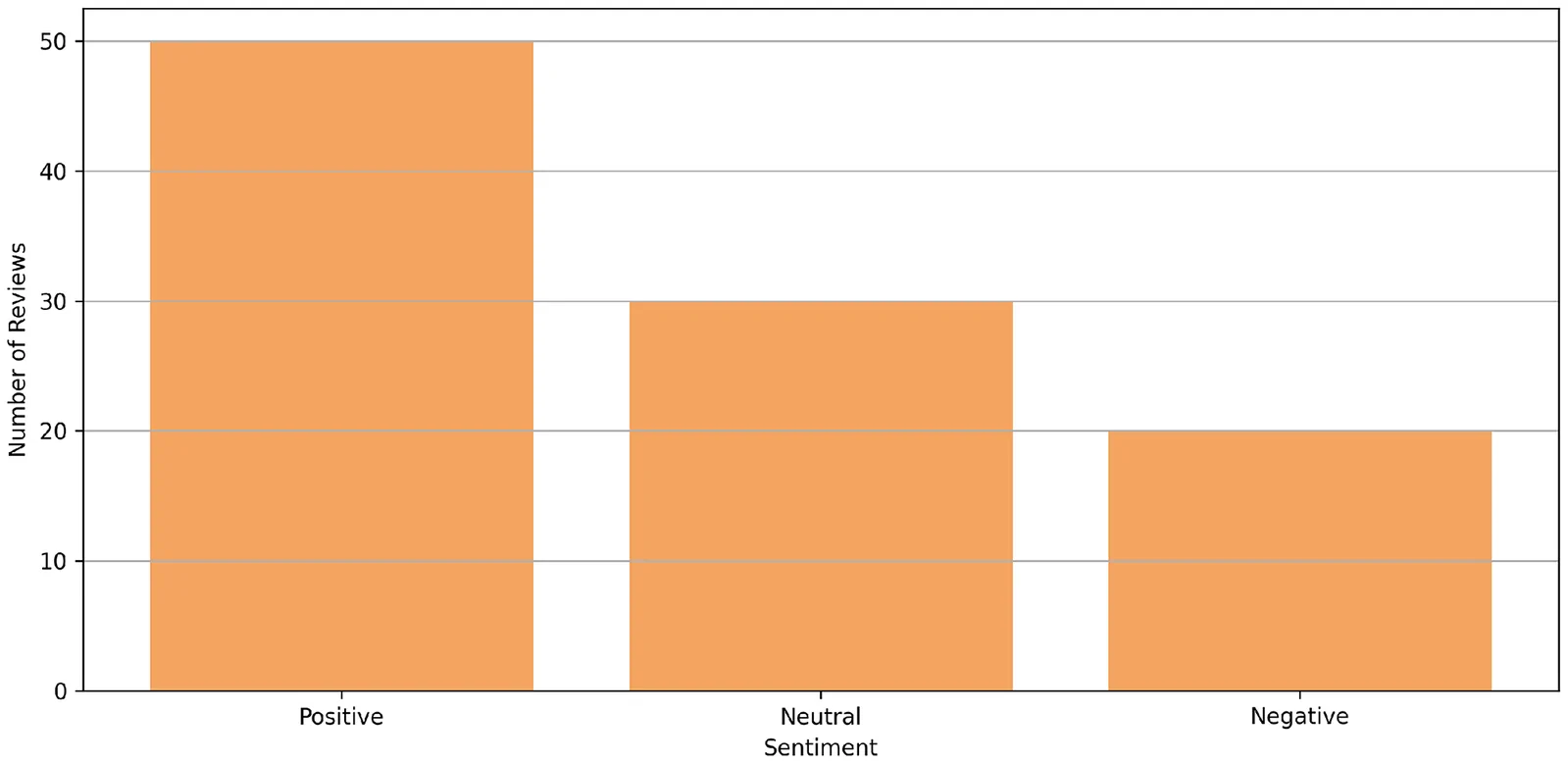
Sentiment analysis results for retrieved reviews

The performance of the review retrieval tool was evaluated based on its ability to retrieve real patient feedback and accurately analyze the sentiment of those reviews. The tool demonstrated strong performance, achieving an accuracy of 97% in categorizing reviews as positive, neutral, or negative. Figure 11 shows a detailed breakdown of the sentiment analysis results, indicating that 50% of the reviews were classified as positive, 30% as neutral, and 20% as negative.

This visualization underscores the tool’s effectiveness in categorizing feedback, providing valuable insights into patient experiences. By identifying sentiment trends, healthcare services can pinpoint areas for improvement and reinforce positive aspects of patient care.

Additionally, the tool’s capability was demonstrated through a specific scenario involving the retrieval of negative hospital reviews:

**Scenario: Retrieving Negative Hospital Reviews**

- **Input:** “What are examples of negative hospital reviews?”
- **Output:** “One example of a negative review is: ‘The hospital staff was friendly and accommodating; however, the lack of a proper feedback system made it difficult to express concerns and suggestions.’”

This example highlights how the tool can extract valuable patient feedback, offering insights that can be used to improve healthcare services by addressing concerns and maintaining high standards of patient care.

### Impact of historical context integration

**Fig. 12:**
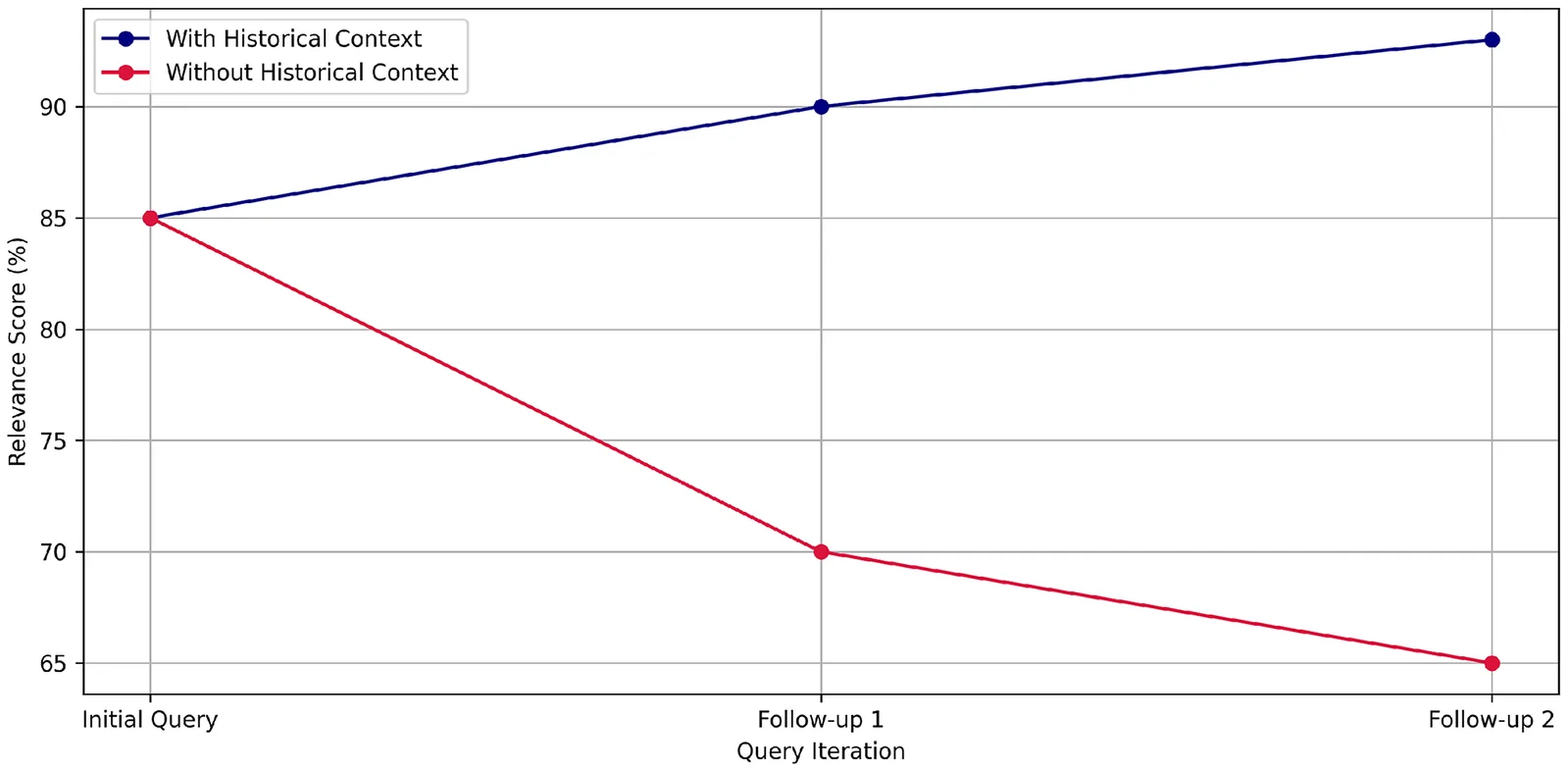
Impact of historical context integration on response relevance

To enhance the coherence and relevance of responses in follow-up queries, a historical context mechanism was integrated into the model. This feature allows the system to consider previous interactions, providing more contextually appropriate responses. As shown in Fig. 12, the inclusion of historical context significantly improved the relevance of responses over multiple iterations of user queries.

For example, while the relevance score remained consistent at 85% for initial queries, follow-up queries without historical context experienced a drop in relevance to 65%. In contrast, when historical context was applied, the relevance score increased to 93%, highlighting the clear benefit of incorporating previous user interactions into the system’s response mechanism.

This improvement is further illustrated in the following scenario:

**Scenario: Impact of Incorporating Historical Context**

- **Initial Query:** “What are the symptoms of PCOS?”
- **Response:** “Polycystic Ovary Syndrome (PCOS) is a common endocrine disorder in women of reproductive age. Symptoms may include irregular or heavy menstrual periods, excess hair growth, acne, pelvic pain, and difficulty getting pregnant.”
- **Follow-up Query:** “What should I do if I have these symptoms?”
- **Output:** “If you’re experiencing symptoms of PCOS, consult a healthcare professional for a proper diagnosis. Maintaining a healthy lifestyle, including regular exercise and a balanced diet, can also help manage symptoms.”

This scenario demonstrates how the use of historical context allows the system to build upon previous responses, providing users with more comprehensive and relevant information, thereby enhancing the overall interaction quality.

### Discussion and insights

The experimental results demonstrate that integrating multiple advanced tools—such as the knowledge graph, Wikipedia tool, symptom checker, and review retrieval mechanism—substantially enhances the system’s ability to deliver precise, relevant, and user-specific medical information. Each tool plays a distinct yet complementary role in addressing different aspects of user queries, leading to a more comprehensive and well-rounded healthcare support system.

The knowledge graph proved particularly valuable in handling structured and complex queries, offering precise, interconnected information about symptoms, diseases, and patient profiles. This tool enabled the system to retrieve detailed relationships between various medical entities, such as identifying symptoms associated with a specific condition or patients displaying certain symptoms. Its ability to accurately process structured data made it an indispensable component for medical decision-making and data retrieval.

The Wikipedia tool, on the other hand, was highly effective in addressing general medical inquiries with comprehensive, up-to-date information. Its ability to access a vast database of medical knowledge ensured that users received detailed, reliable answers to questions about treatments, diagnoses, and medical conditions. The rapid response times of the Wikipedia tool further enhanced the system’s real-time usability, making it an efficient resource for general health information.

The symptom checker provided users with actionable, contextually relevant health advice based on their symptom input. By calculating probabilities for various conditions (such as the common cold, flu, or COVID-19), the tool not only suggested potential diagnoses but also recommended appropriate next steps, such as testing or medical consultation. This feature empowers users to make informed decisions about their health, enhancing the system’s role as a proactive healthcare assistant.

The review retrieval tool added another layer of functionality by analyzing real patient feedback and performing sentiment analysis. This tool’s ability to categorize reviews into positive, neutral, or negative sentiments offered valuable insights into patient experiences and highlighted areas for potential improvement in healthcare services. Its high accuracy in sentiment analysis ensures that feedback is correctly interpreted, making it a useful tool for healthcare providers looking to enhance patient satisfaction.

One of the standout features of the system was the integration of a historical context mechanism, which played a crucial role in maintaining the continuity of user interactions. In healthcare settings, where user queries often build on prior information, this feature ensured that the system provided more context-aware and relevant responses. By leveraging previous interactions, the system could offer coherent, sequential guidance, which is especially important in addressing follow-up medical queries or ongoing health concerns.

The integration of these tools and features has resulted in a more robust, dynamic, and reliable AI medical assistant that is capable of addressing a wide spectrum of healthcare needs. The system’s ability to handle both structured and unstructured queries, coupled with its contextual awareness, significantly enhances patient engagement and satisfaction. Additionally, the system’s capability to deliver personalized, accurate, and timely information makes it a promising solution for practical healthcare applications. This AI-driven platform has the potential to revolutionize patient support by improving access to information, facilitating informed decision-making, and providing ongoing health guidance, thus aligning with the growing demand for intelligent, responsive healthcare systems.

## Limitations

While this study presents an AI-driven medical agent that provides accurate and contextually relevant responses, several limitations should be considered. These limitations primarily concern the data, scalability, and future enhancements of the system.

**1. Data Size and Representativeness:** The dataset used for fine-tuning the model was relatively small, with 1,506,135 training tokens. While this allowed us to test and fine-tune the model effectively, it may not be representative of the vast diversity of medical conditions and patient demographics. The limited scope of the dataset means that the AI agent may not capture all variations in patient profiles, which could affect the generalizability of its responses.

**2. Continuous Fine-Tuning:** As medical knowledge evolves rapidly, continuous fine-tuning of the model is necessary to keep it updated. The current model is based on data available at the time of training, and it may not fully account for newer guidelines, research, or emerging medical conditions. Ongoing fine-tuning is essential to ensure the system remains accurate and relevant over time.

**3. Scalability and Infrastructure:** The current implementation of the system was run on a regular computer. To scale the system for high-demand scenarios, dedicated servers and powerful GPUs are required. The lack of a robust infrastructure could limit the system’s ability to handle large volumes of concurrent user interactions effectively, and scalability remains a key challenge for future deployments.

**4. Multilingual Support:** The current version of the AI agent operates primarily in English, limiting its accessibility for non-English-speaking users. In future iterations, incorporating multilingual translation capabilities will be crucial to expand the reach of the system and make it available to a wider user base.

**5. Multimodal Support:** At present, the system supports text-based interaction only. However, integrating multimodal capabilities such as voice-based interaction or data from wearable health devices would enhance the system’s usability and provide a more holistic view of a patient’s health. These features are essential for improving the overall user experience and ensuring comprehensive patient care.

Despite these limitations, the primary aim of the study was to develop an AI agent capable of delivering precise and accurate medical insights. We achieved this goal, and the aforementioned limitations are areas of future work aimed at enhancing the system’s functionality, scalability, and inclusivity.

## Conclusion and future work

This study introduced an AI-driven medical assistant system that integrates multiple advanced AI tools, including a knowledge graph, Wikipedia API, symptom checker, and review retrieval mechanism, all coordinated by a dynamic query management agent. The results demonstrate that this multi-component approach significantly enhances the system’s ability to provide precise, relevant, and contextually informed medical responses. By dynamically selecting the most suitable tools based on user queries, the system ensures efficient information retrieval and decision support, fostering improved patient engagement and assisting healthcare professionals in making more informed decisions.

This research makes notable contributions to the intersection of AI, healthcare, and information retrieval systems. Theoretically, it extends prior work on intelligent medical assistants by demonstrating how a multi-agent, tool-integrated approach can enhance the contextual relevance and accuracy of medical responses. The incorporation of knowledge graphs for structured reasoning and RAG for real-time information synthesis represents a novel combination that improves medical query handling. Additionally, the study contributes to AI-based healthcare decision support by exploring how dynamic query orchestration optimizes AI resource utilization.

From a practical perspective, the system provides a scalable and adaptable solution for patient self-diagnosis, healthcare decision support, and medical information retrieval. Unlike conventional symptom checkers or static chatbots, this system dynamically integrates real-world patient reviews and external knowledge sources, allowing it to offer more holistic and context-aware recommendations. Furthermore, its ability to maintain conversational history enhances continuity in patient interactions, reducing redundant questioning and improving the overall user experience.

The proposed system holds several practical advantages over existing AI-driven medical assistants. First, it improves medical knowledge accessibility, enabling patients and healthcare providers to obtain reliable, real-time, and well-contextualized medical information. Second, the personalized symptom checker enhances preliminary self-assessment, allowing users to gain health insights before consulting a physician. Additionally, healthcare professionals can leverage the system for rapid knowledge retrieval, assisting them in making informed clinical decisions. Lastly, the integration of real patient reviews provides valuable community-driven insights that could help both patients and doctors gauge treatment efficacy based on real-world experiences.

Despite its strengths, this study has certain limitations. First, the system relies on publicly available medical knowledge sources (such as Wikipedia), which may not always contain the most specialized medical information. While knowledge graphs help structure and validate responses, they are limited by the quality of the underlying datasets. Second, the absence of multimodal data processing (e.g., medical imaging or wearable sensor data) restricts the system’s ability to handle cases where visual diagnosis is crucial. Additionally, while the system provides context-aware responses, it does not yet incorporate long-term patient history or evolving health conditions, which could further improve personalized recommendations. Finally, privacy and ethical concerns surrounding AI-driven medical advice warrant further research, particularly in terms of data security, misinformation risks, and regulatory compliance.

Building on these findings, several promising directions for future research emerge:

- Expanding the Medical Knowledge Base: Incorporating more specialized datasets, clinical trial results, and peer-reviewed medical literature would enhance the system’s reliability and applicability to specific medical domains, such as rare diseases or precision medicine.
- Enhancing Context Awareness and Long-Term Memory: Future iterations should focus on developing patient-specific memory mechanisms that allow the system to track user interactions over time, providing more personalized, history-aware recommendations.
- Integration of Multimodal Data Processing: Incorporating medical imaging analysis, wearable sensor data, and voice-based interactions could significantly improve diagnostic capabilities, making the system more versatile in real-world medical applications.
- Ethical AI and Privacy-Preserving Mechanisms: Addressing privacy concerns through federated learning, homomorphic encryption, and differential privacy techniques would ensure secure, compliant AI-driven medical assistance.
- Clinical Validation and Real-World Deployment: Conducting real-world clinical trials and user studies in hospitals and telemedicine platforms would validate the system’s effectiveness, usability, and trustworthiness in practical healthcare settings.

By addressing these future research directions, the AI-driven medical assistant system can evolve into a more reliable, adaptable, and impactful tool for both patients and healthcare providers, ultimately contributing to improved healthcare accessibility and decision-making.

## Data Availability

Data is provided within the manuscript.

## Abbreviations

AI: Artificial Intelligence
API: Application Programming Interface
EHR: Electronic Health Records
RAG: Retrieval-Augmented Generation
PCOS: Polycystic Ovary Syndrome
NLP: Natural Language Processing
GPT: Generative Pre-trained Transformer
LLM: Large Language Model
ELISA: Enzyme-Linked Immunosorbent Assay
MTS: Medical Text Summarization
IRB: Institutional Review Board
JSON: JavaScript Object Notation
UMLS: Unified Medical Language System
SNOMED CT: Systematized Nomenclature of Medicine Clinical Terms
PMC: PubMed Central
